# Computed Tomography Perfusion and Angiography for Death by Neurologic Criteria

**DOI:** 10.1001/jamaneurol.2025.2375

**Published:** 2025-06-13

**Authors:** Michaël Chassé, Jai Jai Shiva Shankar, Dean A. Fergusson, Shane W. English, Sonny Dhanani, François Lauzier, Alexis F. Turgeon, Ian Ball, Sultan Darvesh, Joel Neves Briard, Marco Essig, David Boucher-Roy, Polina Titova, Martine Lebrasseur, Philippe Couillard, Andreas Kramer, Frédérick D’Aragon, Mathew Hannouche, Donatella Tampieri, Maureen O. Meade, Bijoy K. Menon, Robert Green, Andrew J. Baker, Karen E. A. Burns, Ryan Zarychanski, Jason Shahin, J. Gordon Boyd, Alexandra Binnie, Andrew Gibson, Han Ting Wang, Sam Shemie

**Affiliations:** 1 Department of Medicine, Université de Montréal, Montréal, Québec, Canada; 2 Centre de Recherche du Centre Hospitalier de l’Université de Montréal, Montréal, Québec, Canada; 3 School of Public Health, Université de Montréal, Montréal, Québec, Canada; 4 Department of Radiology, University of Manitoba, Winnipeg, Manitoba, Canada; 5 Department of Human Anatomy and Cell Sciences, Winnipeg, Manitoba, Canada; 6 Methodological and Implementation Research Program, Ottawa Hospital Research Institute, Ottawa, Ontario, Canada; 7 Department of Medicine, University of Ottawa, Ottawa, Ontario, Canada; 8 Division of Critical Care Medicine, Department of Medicine, University of Ottawa, Ottawa, Canada; 9 Acute Care Research, Ottawa Hospital Research Institute, Ottawa, Ontario, Canada; 10 School of Epidemiology and Public Health, University of Ottawa, Ottawa, Ontario, Canada; 11 Department of Pediatrics, University of Ottawa, Ottawa, Ontario, Canada; 12 Division of Critical Care Medicine, Department of Anesthesiology and Critical Care Medicine, Université Laval, Québec City, Québec, Canada; 13 Population Health and Optimal Health Practices Research Unit, Trauma–Emergency–Intensive Care, Centre Hospitalier Universitaire de Québec–Université Laval Research Center, Québec City, Québec, Canada; 14 Department of Medicine, Université Laval, Québec City, Québec, Canada; 15 Department of Medicine, Western University, London, Ontario, Canada; 16 Department of Epidemiology and Biostatistics, Western University, London, Ontario, Canada; 17 Department of Medicine, Dalhousie University, Halifax, Nova Scotia, Canada; 18 Department of Medical Neuroscience, Dalhousie University, Halifax, Nova Scotia, Canada; 19 Department of Neuroscience, Université de Montréal, Montréal, Québec, Canada; 20 Department of Critical Care Medicine, University of Calgary, Calgary, Alberta, Canada; 21 Department of Clinical Neurosciences, Hotchkiss Brain Institute, University of Calgary, Calgary, Alberta, Canada; 22 Give Life Alberta, Edmonton, Alberta, Canada; 23 Department of Anesthesiology, Université de Sherbrooke, Sherbrooke, Québec, Canada; 24 Centre de Recherche du Centre Hospitalier Universitaire de Sherbrooke, Sherbrooke, Québec, Canada; 25 Department of Medicine, McGill University, Montréal, Québec, Canada; 26 Department of Radiology, Queen’s University, Kingston, Ontario, Canada; 27 Division of Critical Care, Department of Medicine, McMaster University, Hamilton, Ontario, Canada; 28 Department of Radiology, Hotchkiss Brain Institute, University of Calgary, Calgary, Alberta, Canada; 29 Department of Community Health Sciences, Hotchkiss Brain Institute, University of Calgary, Calgary, Alberta, Canada; 30 Department of Critical Care, Dalhousie University, Halifax, Nova Scotia, Canada; 31 Department of Emergency Medicine, Dalhousie University, Halifax, Nova Scotia, Canada; 32 Department of Anesthesia, Dalhousie University, Halifax, Nova Scotia, Canada; 33 Department of Surgery, Dalhousie University, Halifax, Nova Scotia, Canada; 34 Interdepartmental Division of Critical Care, Unity Health Toronto–St Michael’s Hospital, Toronto, Ontario, Canada; 35, Section of Critical Care, Department of Internal Medicine, Max Rady College of Medicine, University of Manitoba, Winnipeg, Manitoba, Canada; 36 Department of Medical Oncology and Hematology, CancerCare Manitoba, Winnipeg, Manitoba, Canada; 37 Section of Hematology/Medical Oncology, Department of Internal Medicine, Max Rady College of Medicine, University of Manitoba, Winnipeg, Manitoba, Canada; 38 Critical Care Program, Department of Medicine, McGill University Health Centre, Montréal, Québec, Canada; 39 Division of Neurology, Department of Medicine, Queen’s University, Kingston, Ontario, Canada; 40 Department of Critical Care Medicine, Queen’s University, Kingston, Ontario, Canada; 41 Department of Critical Care, William Osler Health System, Toronto, Ontario, Canada; 42 Division of Critical Care Medicine, Department of Medicine, Maisonneuve-Rosemont Hospital, Montréal, Québec, Canada; 43 Division of Critical Care Medicine, Montreal Children’s Hospital, McGill University, Montréal, Québec, Canada

## Abstract

**Importance:**

Accurate and timely confirmation of death by neurologic criteria (DNC) is essential for clinical decision-making and organ-donation processes, yet currently available ancillary tests have suboptimal diagnostic performance or limited validation.

**Objectives:**

To determine the diagnostic accuracy, interrater reliability, and safety of brain computed tomography (CT) perfusion and CT angiography as ancillary investigations for DNC.

**Design, Setting, and Participants:**

Between April 25, 2017, and March 10, 2021, a prospective, multicenter, blinded diagnostic accuracy cohort study was conducted in 15 adult intensive care units across Canada. Consecutive, critically ill adults (aged ≥18 years) with a Glasgow Coma Scale score of 3 and no confounding factors who were at high risk of DNC were included. Data collection and analysis were performed from April 2021 to July 2024.

**Exposure:**

Contrast-enhanced brain CT perfusion with CT angiography reconstructions performed within 2 hours of a blinded, standardized clinical DNC examination.

**Main Outcomes and Measures:**

The primary outcomes were the sensitivity and specificity of qualitative and quantitative brainstem CT perfusion for DNC determination, assessed by 2 independent neuroradiologists blinded to clinical findings; the prespecified validation threshold was greater than 98%. Secondary outcomes were the diagnostic accuracy of whole-brain CT perfusion and CT angiography, interrater reliability (Cohen κ), and adverse events associated with imaging.

**Results:**

A total of 282 patients (mean [SD] age, 57.8 [15.4] years; 133 [47%] female) completed the study protocol and were included in the primary analysis; 204 (72%) of these were ultimately declared deceased by standardized clinical criteria. Qualitative brainstem CT perfusion showed a sensitivity of 98.5% (95% CI, 95.8%-99.7%) and a specificity of 74.4% (95% CI, 63.2%-83.6%); quantitative brainstem CT perfusion was not diagnostically accurate. Qualitative whole-brain CT perfusion yielded a sensitivity of 93.6% (95% CI, 89.3%-96.6%) and a specificity of 92.3% (95% CI, 84.0%-97.1%). CT angiography sensitivity ranged from 75.5% (95% CI, 69.0%-81.2%) to 87.3% (95% CI, 81.9%-91.5%), and its specificity ranged from 89.7% (95% CI, 80.8%-95.5%) to 91.0% (95% CI, 82.4%-96.3%). Interrater reliability was excellent for all ancillary tests (κ ranged from 0.81 [95% CI, 0.73-0.89] to 0.84 [95% CI, 0.78-0.91]). Fourteen patients (5%) experienced minor, self-limited adverse events; no serious adverse events occurred.

**Conclusions and Relevance:**

The observed sensitivity and specificity measures for CT perfusion and CT angiography as an ancillary test for DNC did not meet the prespecified validation threshold of greater than 98%. Clinical examination remains the cornerstone of DNC, and ancillary imaging should be interpreted cautiously within a comprehensive clinical assessment.

## Key Points

**Question:**

In intensive care unit patients at risk of death by neurologic criteria (DNC), what is the diagnostic accuracy of brain computed tomography (CT) perfusion and CT angiography compared with the clinical examination?

**Findings:**

In a multicenter diagnostic study of 282 patients (204 with DNC), qualitative brainstem CT perfusion showed 98.5% sensitivity and 74.4% specificity, whole-brain CT perfusion showed 93.6% sensitivity and 92.3% specificity, and CT angiography showed 75.5% to 87.3% sensitivity and 89.7% to 91.0% specificity.

**Meaning:**

None of the imaging modalities met the prespecified accuracy threshold of greater than 98%, supporting their role only as ancillary rather than standalone tests for confirming DNC.

## Teaser

This diagnostic study evaluates the diagnostic accuracy, interrater reliability, and safety of brain computed tomography perfusion and computed tomography angiography for death by neurologic criteria.

## Introduction

Determination of death by neurologic criteria (DNC) is a critical aspect of modern medicine, defining the end of life for many individuals with acute brain injury. DNC is defined by the permanent cessation of brain function and is characterized by the complete absence of consciousness and of brainstem reflexes, including the capacity to breathe, following a catastrophic brain injury.^1,2,3^ False-negative DNC may prolong the provision of futile organ support, and false-positive DNC may lead to an erroneous declaration of death. Although clinical evaluation forms the cornerstone of DNC, its validity can be influenced by confounding factors, such as the effects of sedative medications, traumatic injuries to the skull, or severe metabolic derangements. In these circumstances, DNC guidelines recommend additional ancillary investigation to determine the absence of brain blood flow or perfusion.^1,2,3^

Nevertheless, ancillary investigations do not directly assess pertinent clinical function.^4^ Significant variability exists in physician preferences^5,6,7^ and in hospital policies, national regulatory committees, or law^8,9^ regarding their indications and use. A recent systematic review concluded that studies assessing the diagnostic accuracy of DNC ancillary investigations are at risk of bias with variable or unclear sensitivities and specificities that could lead to misclassifications.^10^ Despite the widespread use of 4-vessel conventional angiography as a DNC ancillary investigation, the systematic review^10^ and the World Brain Death Project consensus statement^2^ acknowledged that the specificity of 4-vessel conventional angiography is unknown due to lack of thorough investigation in an appropriate diagnostic test accuracy study. The World Brain Death Project identified the diagnostic accuracy of novel DNC ancillary investigations, such as CT perfusion and CT angiography, as a knowledge gap and research priority in the field of death determination.^2^ Whereas CT angiography visualizes blood flow within large cerebral vessels, CT perfusion assesses tissue-level microvascular perfusion.^11,12^ Importantly, neither technique measures neuronal function itself, which is what is assessed clinically or with functional tests, such as electroencephalography or evoked potentials. Preliminary studies have indicated CT perfusion’s potential as a DNC ancillary investigation, but adequate validation is necessary.^13^

The primary objective of this study was to evaluate the diagnostic accuracy of brainstem CT perfusion for DNC based on qualitative and quantitative assessments. Secondary objectives were to assess the diagnostic accuracy of whole-brain CT perfusion and of CT angiography for DNC and to determine the safety and consistency of CT perfusion and CT angiography in critically ill patients.

## Methods

In collaboration with the Canadian Critical Care Trials Group, we conducted a prospective multicenter diagnostic accuracy study in 15 Canadian academic intensive care units (eAppendix 1 in Supplement 1). We obtained research ethics board approval at each participating site and written informed consent from each patient’s surrogate decision-maker prior to enrollment. The study was overseen by a steering committee composed of experts in critical care, neurology, and neuroradiology. Study reporting follows the Standards for Reporting of Diagnostic Accuracy (STARD) reporting guidelines.^14^ Data collection and analysis were performed from April 2021 to July 2024.

### Study Population and Eligibility Criteria

Demographic characteristics, such as sex and race (reported by surrogate decision-makers or recorded in hospital records as Asian, Black or African American, First Nations, White, and other [Arab, Latin American, and multiracial, grouped owing to small sample sizes] or unknown [declined to report or could not be determined by the study team]), were collected per protocol at the time of enrollment to describe the included population and to inform the generalizability of our findings. To effectively evaluate the diagnostic accuracy (sensitivity and specificity) of CT perfusion and DNC, the study was designed to carefully include patients both with and without a final diagnosis of DNC. This mixed sample was essential to determine the true-positive rates (sensitivity), false-negative rates, true-negative rates (specificity), and false-positive rates. Therefore, our selection criteria were designed to enroll patients suspected of DNC, specifically encompassing individuals presumed to be at high risk of progressing to DNC. To ensure a reliable evaluation of diagnostic accuracy, we also implemented strict exclusion criteria to remove factors that could interfere with or confound the clinical diagnosis of DNC. We screened adults aged 18 years or older who were admitted to the intensive care unit with acute and devastating brain injury confirmed by neuroimaging and in whom the Glasgow Coma Scale score was 3 despite cessation of sedation for at least 6 hours. We excluded patients with contraindications to CT perfusion, hemodynamic instability that prevented safe transport to the CT scanner, and patients with any confounding factors that could have precluded a reliable clinical evaluation (eAppendix 2 in Supplement 1). Eligibility of all enrolled patients was confirmed by blind central adjudication (eAppendix 2 in Supplement 1).

### Ancillary Investigation

Enrolled patients underwent whole-brain CT perfusion ensuring coverage of at least 8 cm starting at the foramen magnum (eAppendix 2 in Supplement 1). Unlike standard clinical practice, in which ancillary tests are typically performed after completion of the full clinical examination, imaging was conducted prior to the formal DNC. This timing was chosen to ensure the shortest possible interval between the ancillary investigation and the reference standard, while also allowing for real-time cancellation or rescheduling of imaging when clinically appropriate, balancing study feasibility with the realities of critical care practice. A total of 40 mL of nonionic iodinated contrast media was injected intravenously. Each study center transferred unprocessed CT perfusion source images to a central imaging core laboratory for processing and analysis. Image processing was performed using Olea Sphere version 3.0 (Olea Medical). CT angiography images were derived from the CT perfusion images using reconstructions, with a peak phase and a late-phase image defined on the time density curve. CT perfusion and CT angiography images were interpreted independently by 2 experienced neuroradiologists blinded to all clinical information, including results from the DNC clinical examination.

For qualitative CT perfusion interpretation, images were assessed for a matched decrease in cerebral blood flow and a corresponding decrease in cerebral blood volume. For qualitative brainstem CT perfusion, the test result was judged positive based on brainstem assessment alone, regardless of findings in the rest of the brain. For whole-brain CT perfusion, the test result was considered positive based on assessment of the entire brain. For quantitative brainstem CT perfusion, large regions of interest were assessed on axial slices at the medulla, pons, and midbrain, and the test result was judged positive if cerebral blood flow was below 10 mL/100 g/min and if cerebral blood volume was below 2 mL/100 g on at least 2 consecutive 5-mm axial slices of brainstem.^12,13^ For CT angiography, neuroradiologists assessed different segments of intracranial arteries on 3 standardized scales (10-point, 7-point, and 4-point scales), each applied separately to the early- and late-acquisition phases.^11,15,16^ Imaging interpretation disagreements were resolved by consensus using a third reading, blinded from the first 2 interpretations and DNC clinical examination result.

### Determination of DNC

Within 2 hours of ancillary investigation, 2 clinicians (neurologists, neurosurgeons, or intensivists) performed a complete clinical DNC assessment. Both clinicians used standardized criteria based on contemporary DNC guidelines^17,18^ and were blinded to the results of the study’s ancillary investigation. Patients were declared deceased by neurologic criteria when the following criteria were met: (1) documentation of a devastating brain injury with an established etiology and plausibility of causing DNC; (2) absence of confounders that could affect the reliability of the clinical neurologic examination (including measure of plasma concentrations of common sedatives and analgesics^19^); (3) absence of brainstem reflexes and motor response to central pain; and (4) positive apnea test result (eAppendix 2 in Supplement 1). Patients were classified as clinically deceased by neurologic criteria, or not, by 2 examining physicians. For this study, the clinical neurologic evaluation was considered the reference standard for DNC.

### Outcome Measures

For all patients, detailed demographic characteristics, clinical information, scan-related adverse events, and clinical outcomes were collected. Because clinical DNC principally focuses on brainstem function (absence of brainstem reflexes and respiratory drive), our primary outcome was the diagnostic accuracy of brainstem perfusion assessment by CT perfusion. Secondary outcomes were diagnostic accuracy of whole-brain CT perfusion and of CT angiography, as well as their safety and consistency (interrater reliability). Ancillary investigation results were considered a true positive or a true negative when concordant with the reference standard. False-positive or false-negative results occurred when discordant with the reference standard.

### Statistical Analysis

We estimated that 45% to 55% of eligible patients would satisfy bedside-determined DNC. Under this prevalence, enrolling 270 protocol-complete patients would allow estimation of brainstem CT perfusion sensitivity and specificity of greater than 98% with a margin of error of 2.5% or less at an α error of .05.

To account for unforeseen events during the study procedure, the initial expected enrollment sample size was 300. In August 2020, blinded progress reports showed a higher-than-expected rate of protocol noncompletion, so the executive committee increased the enrollment target to 330 to ensure that the planned 270 protocol-complete patients would be achieved.

We only included patients with complete CT perfusion evaluations and a clinical evaluation without confounding, as determined by adjudication of the clinical files and blinded to the imaging results. Baseline characteristics were assessed using frequency distributions. For primary analyses, we calculated qualitative and quantitative brainstem CT perfusion sensitivities, specificities, positive predictive values, negative predictive values, positive likelihood ratios, and negative likelihood ratios with corresponding 95% CIs. For secondary analyses, we assessed the respective diagnostic accuracy of qualitative whole-brain CT perfusion and of CT angiography using the same steps as brainstem CT perfusion. We also evaluated interrater agreement for each ancillary investigation using the Cohen κ statistic, and we reported descriptive frequency distribution statistics to describe safety adverse events. R version 4.3.3 software (R Foundation) was used for statistical analysis.

In preplanned subgroup analyses, we assessed diagnostic accuracy of the ancillary investigations based on age (<60 vs ≥60 years), sex (male, female), type of brain injury (traumatic brain injury, anoxic brain injury, ischemic stroke, hemorrhagic stroke, other), and presence or absence of artifacts on ancillary imaging.

We conducted 2 preplanned sensitivity analyses. First, we excluded patients without a reference standard consensus and patients who had protocol violations. Second, we tested for potential death misclassification in the reference standard by excluding patients who were considered alive based on peripheral movements alone.

## Results

**Figure 1.**
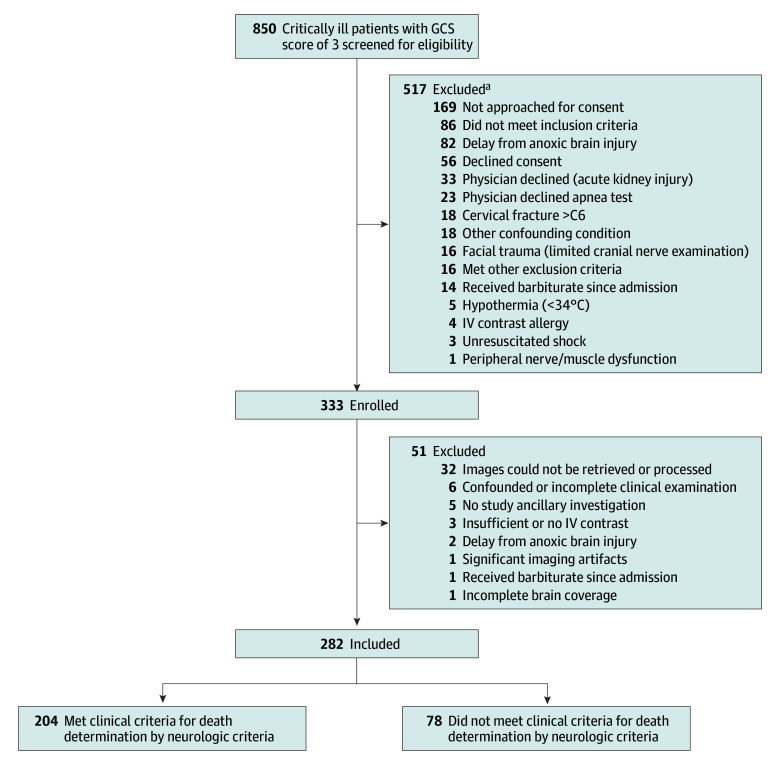
Study Flowchart GCS indicates Glasgow Coma Scale; IV, intravenous. ^a^The reasons for these exclusions are not mutually exclusive.

Between April 25, 2017, and March 10, 2021, 333 patients were enrolled, of whom 282 were included in the final analysis (Figure 1 and Table 1). Exclusion of 51 enrolled patients occurred primarily due to incomplete or confounded clinical examinations, technical issues with CT perfusion, or withdrawal of consent. Mean (SD) age was 57.8 (15.4) years, and 133 patients (47%) were female. Causes of brain injury were hemorrhagic stroke in 150 patients (53%), traumatic brain injury in 40 (14%), anoxic brain injury in 59 (21%), ischemic stroke in 15 (5%), tumor in 1 (0.4%), infection in 4 (1%), and other causes in 13 (5%). Clinical DNC assessments occurred a median (IQR) of 64.5 (40.0-87.8) minutes after ancillary testing. Central adjudication confirmed that none of the patients included in the analyses were confounded by sedative medications based on the plasma concentrations of common sedatives. Based on the clinical neurologic evaluation (reference standard), 204 patients (72%) were determined to be deceased by neurologic criteria (Table 2; eTables 1-3 in Supplement 1), with the remaining 78 (28%) classified as being alive.

**Table 1.** Baseline Characteristics

Characteristic	Total (N = 282)	Deceased (n = 204)^a^	Alive (n = 78)^a^
Age, mean (SD), y	57.8 (15.4)	57.3 (16.1)	59.1 (13.6)
Sex, No. (%)			
Female	133 (47)	110 (54)	23 (29)
Male	149 (53)	94 (46)	55 (71)
Race, No. (%)			
Asian	24 (9)	20 (10)	4 (5)
Black or African American	4 (1)	3 (2)	1 (1)
First Nations	11 (4)	10 (5)	1 (1)
White	217 (77)	153 (75)	64 (82)
Other or unknown^b^	26 (9)	18 (9)	8 (10)
Cause of brain injury, No. (%)			
Traumatic brain injury	40 (14)	28 (14)	12 (15)
Hemorrhagic stroke	150 (53)	121 (59)	29 (37)
Ischemic stroke	15 (5)	13 (6)	2 (3)
Anoxic brain injury	59 (21)	31 (15)	28 (36)
Tumor	1 (0.4)	0	1 (1)
Infection	4 (1)	4 (2)	0
Other	13 (5)	7 (3)	6 (8)
Weight at admission, mean (SD), kg	80.5 (21.0)	79.1 (21.8)	83.9 (18.6)
Comorbidity, No. (%)			
Hypertension treated with medication	86 (31)	59 (29)	27 (34)
Diabetes	50 (18)	30 (15)	20 (26)
Coronary artery disease	40 (14)	23 (11)	17 (22)
Peripheral vascular disease	6 (2)	4 (2)	2 (3)
Previous stroke	27 (10)	19 (9)	8 (10)
Active smoking	42 (15)	28 (14)	14 (18)
Chronic kidney failure			
With dialysis, No. (%)	3 (1)	0	3 (4)
Without dialysis, No. (%)	10 (4)	7 (3)	3 (4)
Liver disease	25 (9)	15 (7)	10 (13)
Other	160 (57)	114 (56)	46 (59)
Time between injury and study enrollment, median (IQR), h	48.8 (25.3-87.8)	43.8 (24.4-72.9)	77.2 (33.7-107.0)

^a^ Deceased or alive as per the reference standard (clinical examination for death by neurologic criteria).

^b^ Other or unknown includes participants identified as Arab, Latin American, or multiracial by surrogate decision-makers—or, when no surrogate was available, as recorded in hospital records—along with individuals who declined to report race and ethnicity or whose race and ethnicity could not be determined by the study team. These were grouped owing to small sample size.

**Table 2.** Results From Clinical Examination and Ancillary Investigation

Outcome	No. (%)^a^		
	Total (N = 282)	Deceased (n = 204)^b^	Alive (n = 78)^b^
Reference standard			
Aware of study ancillary investigation prior to clinical evaluation	3 (1)	2 (1)	1 (1)
Aware of other ancillary investigation result prior to clinical evaluation	18 (6)	13 (6)	5 (6)
Ancillary investigation			
Artifacts	62 (22)	53 (26)	9 (11)
Qualitative CT perfusion result			
Brainstem			
Compatible with death	221 (78)	201 (99)	20 (26)
Not compatible with death	61 (22)	3 (1)	58 (74)
Whole brain			
Compatible with death	197 (70)	191 (94)	6 (8)
Not compatible with death	85 (30)	13 (6)	72 (92)
Quantitative CT perfusion result			
Midbrain blood flow <10 mL/100 g/min			
Compatible with death	121 (43)	89 (44)	32 (41)
Not compatible with death	157 (56)	113 (55)	44 (56)
Missing data	4 (1)	2 (1)	2 (3)
Midpons blood flow <10 mL/100 g/min			
Compatible with death	116 (41)	85 (42)	31 (40)
Not compatible with death	162 (57)	117 (57)	45 (58)
Missing data	4 (1)	2 (1)	2 (3)
Medulla blood flow <10 mL/100 g/min			
Compatible with death	102 (36)	77 (38)	25 (32)
Not compatible with death	176 (62)	125 (61)	51 (65)
Missing data	4 (1)	2 (1)	2 (2)
Midbrain blood volume <2 mL/100 g			
Compatible with death	175 (62)	151 (74)	24 (30)
Not compatible with death	103 (36)	51 (25)	52 (66)
Missing data	4 (1)	2 (1)	2 (3)
Midpons blood volume <2 mL/100 g			
Compatible with death	173 (61)	147 (72)	26 (33)
Not compatible with death	105 (37)	55 (27)	50 (64)
Missing data	4 (1)	2 (1)	2 (3)
Medulla blood volume <2 mL/100 g			
Compatible with death	175 (62)	153 (75)	22 (28)
Not compatible with death	103 (37)	49 (24)	54 (69)
Missing data	4 (1)	2 (1)	2 (3)
CT angiography result			
Peak arterial phase			
4-Point scale			
Compatible with death	186 (66)	178 (87)	8 (10)
Not compatible with death	96 (34)	26 (13)	70 (90)
7-Point scale			
Compatible with death	178 (63)	170 (83)	8 (10)
Not compatible with death	104 (36)	34 (17)	70 (90)
10-Point scale			
Compatible with death	174 (62)	167 (82)	7 (9)
Not compatible with death	108 (38)	37 (18)	71 (91)
Late arterial phase			
4-Point scale			
Compatible with death	180 (64)	172 (84)	8 (10)
Not compatible with death	102 (36)	32 (16)	70 (90)
7-Point scale			
Compatible with death	163 (58)	155 (76)	8 (10)
Not compatible with death	119 (42)	49 (24)	70 (90)
10-Point scale			
Compatible with death	161 (57)	154 (76)	7 (9)
Not compatible with death	121 (43)	50 (25)	71 (91)

Abbreviation: CT, computed tomography.

^a^ Totals for some categories may exceed 100% because each percentage is calculated independently and rounded to the nearest whole number.

^b^ Deceased or alive as per the reference standard (clinical examination for death by neurologic criteria).

Protocol violations occurred in 14 patients (5%) (eTable 4 in Supplement 1). Clinicians performing DNC evaluation were aware of results from other ancillary investigations for 18 patients (6% of deceased patients and 6% of living patients) and were mistakenly unblinded to the study CT perfusion scan for 3 patients (1% of deceased patients and 1% of living patients). The delay between ancillary investigations and clinical examinations exceeded 2 hours for 12 patients (4%). Patient clinical outcomes at hospital discharge are provided in eTable 5 in Supplement 1. No patient declared deceased by neurologic criteria was alive at hospital discharge. Six patients (8%) classified as alive eventually survived to hospital discharge.

**Figure 2.**
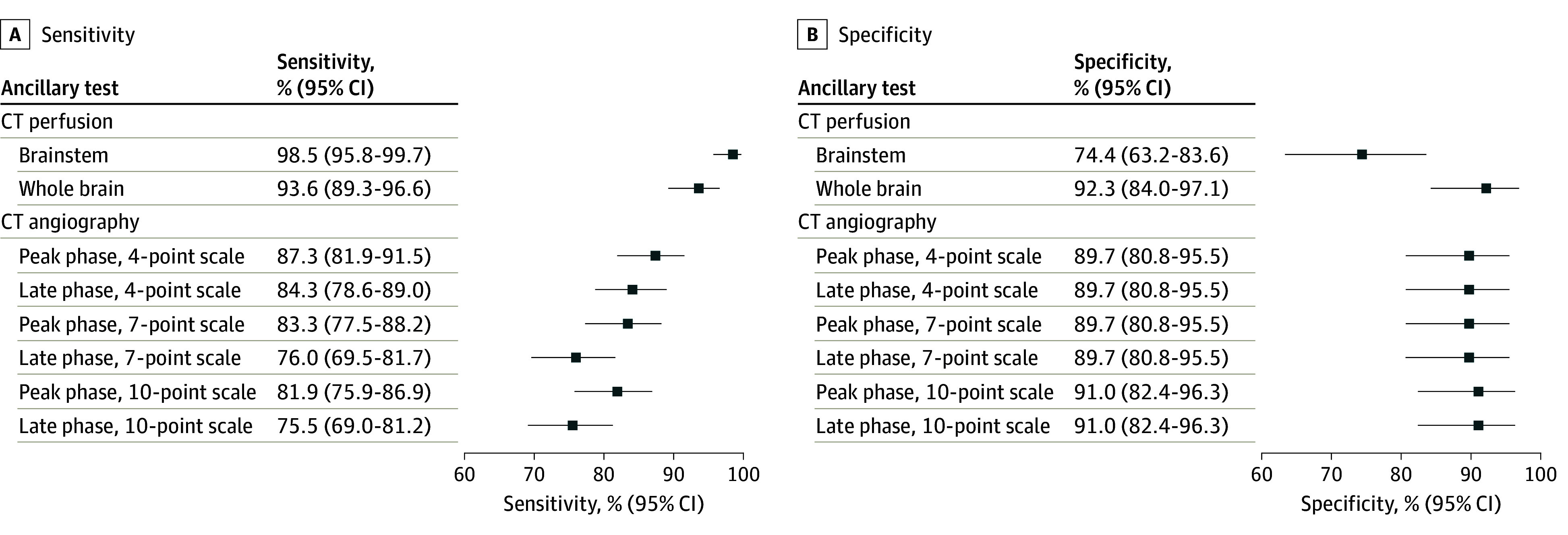
Sensitivity and Specificity of Computed Tomography (CT) Perfusion and of CT Angiography for Death by Neurologic Criteria

Qualitative brainstem CT perfusion had a sensitivity of 98.5% (95% CI, 95.8%-99.7%) and a specificity of 74.4% (95% CI, 63.2%-83.6%) (Figure 2). Quantitative brainstem CT perfusion yielded poor sensitivity and specificity estimates. Qualitative whole-brain CT perfusion had a sensitivity of 93.6% (95% CI, 89.3%-96.6%) and a specificity of 92.3% (95% CI, 84.0%-97.1%). CT angiography sensitivity ranged from 81.9% (95% CI, 75.9%-86.9%) to 87.3% (95% CI, 81.9%-91.5%) for peak-phase examinations and from 75.5% (95% CI, 69.0%-81.2%) to 84.3% (95% CI, 78.6%-89.0%) for late-phase examinations, and specificity ranged from 89.7% (95% CI, 80.8%-95.5%) to 91.0% (95% CI, 82.4%-96.3%) for peak-phase and late-phase examinations. The Cohen κ for interrater reliability was 0.81 (95% CI, 0.73-0.89) for brainstem qualitative CT perfusion, 0.82 (95% CI, 0.74-0.89) for whole-brain qualitative CT perfusion, and 0.82 (95% CI, 0.75-0.89) to 0.84 (95% CI, 0.78-0.91) for CT angiography (Table 3). CT perfusion–related adverse events occurred in 14 patients (5%), with no serious adverse events (eTable 6 in Supplement 1).

**Table 3.** Ancillary Investigation Diagnostic Accuracy and Interrater Reliability

Ancillary investigation	Sensitivity, % (95% CI)	Specificity, % (95% CI)	Accuracy, % (95% CI)	Positive predictive value, % (95% CI)	Negative predictive value, % (95% CI)	Positive likelihood ratio (95% CI)	Negative likelihood ratio (95% CI)	Cohen κ (95% CI)
**Primary outcomes**								
Brainstem CT perfusion								
Qualitative brainstem CT perfusion	98.5 (95.8-99.7)	74.4 (63.2-83.6)	91.8 (88.0-94.8)	91.0 (86.4-94.4)	95.1 (86.3-99.0)	3.84 (2.63-5.61)	0.02 (0.01-0.06)	0.81 (0.73-0.89)
Quantitative CT perfusion								
Midbrain	38.1 (31.4-45.2)	81.6 (71.0-89.5)	50.0 (44.0-56.0)	84.6 (75.5-91.3)	33.2 (26.5-40.4)	2.07 (1.25-3.43)	0.76 (0.65-0.88)	NA
Midpons	36.1 (29.5-43.2)	81.6 (71.0-89.5)	48.6 (42.5-54.6)	83.9 (74.5-90.9)	32.5 (25.9-39.6)	1.96 (1.18-3.26)	0.78 (0.67-0.91)	NA
Medulla	32.7 (26.3-39.6)	90.8 (81.9-96.2)	48.6 (42.5-54.6)	90.4 (81.2-96.1)	33.7 (27.2-40.6)	3.55 (1.70-7.38)	0.74 (0.66-0.84)	NA
**Secondary outcomes**								
Qualitative whole-brain CT perfusion	93.6 (89.3-96.6)	92.3 (84.0-97.1)	93.3 (89.7-95.9)	97.0 (93.5-98.9)	84.7 (75.3-91.6)	12.17 (5.64-26.28)	0.07 (0.04-0.12)	0.82 (0.74-0.89)
Peak-phase CT angiography								
4-Point scale	87.3 (81.9-91.5)	89.7 (80.8-95.5)	87.9 (83.6-91.5)	95.7 (91.7-98.1)	72.9 (62.9-81.5)	8.51 (4.40-16.44)	0.14 (0.10-0.20)	0.84 (0.77-0.90)
7-Point scale	83.3 (77.5-88.2)	89.7 (80.8-95.5)	85.1 (80.4-89.1)	95.5 (91.3-98.0)	67.3 (57.4-76.2)	8.13 (4.20-15.71)	0.19 (0.14-0.25)	0.84 (0.78-0.91)
10-Point scale	81.9 (75.9-86.9)	91.0 (82.4-96.3)	84.4 (79.6-88.4)	96.0 (91.9-98.4)	65.7 (56.0-74.6)	9.12 (4.49-18.55)	0.20 (0.15-0.27)	0.82 (0.75-0.89)
Late-phase CT angiography								
4-Point scale	84.3 (78.6-89.0)	89.7 (80.8-95.5)	85.8 (81.2-89.7)	95.6 (91.4-98.1)	68.6 (58.7-77.5)	8.22 (4.25-15.89)	0.17 (0.13-0.24)	0.83 (0.78-0.90)
7-Point scale	76.0 (69.5-81.7)	89.7 (80.8-95.5)	79.8 (74.6-84.3)	95.1 (90.6-97.9)	58.8 (49.4-67.8)	7.41 (3.83-14.45)	0.27 (0.21-0.35)	0.84 (0.78-0.90)
10-Point scale	75.5 (69.0-81.2)	91.0 (82.4-96.3)	79.8 (74.6-84.3)	95.7 (91.2-98.2)	58.7 (49.4-67.6)	8.41 (4.13-17.13)	0.27 (0.21-0.35)	0.84 (0.78-0.90)

Abbreviation: NA, not applicable.

Twenty patients who did not fulfill clinical criteria for DNC had qualitative brainstem CT perfusion compatible with death (false-positive results; eTable 7 in Supplement 1), whereas 3 patients who were declared deceased clinically had brainstem perfusion (false-negative results; eTable 8 in Supplement 1). When qualitatively assessing the whole brain by CT perfusion, 6 patients who did not fulfill clinical criteria for DNC had whole-brain CT perfusion compatible with death (false-positive results; eTable 9 in Supplement 1), whereas 13 patients who were declared deceased clinically had brain perfusion (false-negatives results; eTable 10 in Supplement 1). Age, sex, type of brain injury, presence of artifacts, and sensitivity analyses were not significantly associated with outcome measures (eFigures 1-4 and eTable 11 in Supplement 1).

## Discussion

In this prospective, multicenter diagnostic accuracy study of patients with a high pretest probability of meeting DNC, brainstem CT perfusion, whether evaluated quantitatively or qualitatively, was not specific for DNC, leading to a noteworthy risk of false-positive death determinations. Qualitative whole-brain CT perfusion provided high but imperfect sensitivity and specificity for DNC. CT angiography demonstrated comparable specificity to CT perfusion but lower sensitivity, increasing the chance of false-negative classifications without reducing the risk of false-positive results. Both ancillary investigations had excellent interrater reliability.

Current DNC guidelines focus on demonstrating permanent loss of brain function, including respiratory drive. However, some jurisdictions also expect demonstration of global intracranial circulatory arrest, especially in the presence of confounding factors or clinical uncertainty. This nuance matters clinically, as a patient with no demonstrable clinical brainstem activity may still have partial blood flow in the brain, making the choice of ancillary test critical. Our findings suggest that qualitative whole-brain CT perfusion poses the lowest risk of falsely classifying a patient who meets bedside DNC criteria as alive, while its risk of misclassifying a living patient as deceased is comparable to that of CT angiography.

These results support the potential role of CT perfusion and CT angiography as ancillary investigations for DNC when such a test is indicated in the comprehensive evaluation of death in a patient with a devastating brain injury. However, imperfect test specificity reinforces the importance of thoroughly evaluating patients’ neurologic status through history and clinical examination prior to conducting ancillary investigations, such as in circumstances where confounding factors cannot be eliminated or a complete clinical examination is not possible. This maximizes ancillary investigation pretest probability, which subsequently increases posttest probability, and minimizes the risk of false-positive death determination. Despite a uniform imaging protocol and postprocessing software, minor scanner-specific variations likely occurred and automated software underperformed relative to expert qualitative reads, consistent with standard neuroimaging practice. Diagnostic accuracy may therefore differ on other CT perfusion platforms that use different perfusion algorithms. Likewise, our CT angiography results were reconstructed from the CT perfusion source data rather than from a dedicated CT angiography protocol, which may potentially influence performance estimates. Finally, our data support previous observations that a subset of patients fulfilling clinical DNC can exhibit residual intracranial blood flow or perfusion.^10,20^ Intracranial vessel opacification was observed in up to 25% of patients meeting clinical DNC. However, cerebral perfusion was observed in only 6% of patients, demonstrating that blood flow in intracranial arteries does not systematically infer capillary-level perfusion.^4^ These observations emphasize the need for a comprehensive approach for accurate and ethical decision-making in DNC that integrates robust clinical evaluation, neuroimaging findings, and understanding of the patient’s overall condition.^20^

The strengths of our study include a clinically relevant heterogeneous sample of patients with brain injury in diverse hospital settings with a study design that permitted robust evaluation of sensitivity and specificity. We minimized biases by using duplicate, fully blinded assessments: clinicians evaluating DNC were blinded to imaging results, and neuroradiologists interpreting the scans were blinded to clinical data. The delay between imaging and clinical assessments was intentionally short, leading to a more accurate assessment of the diagnostic validity of ancillary investigations. We also observed excellent interrater agreement, further supporting the robustness of our results.

### Limitations

This study has several limitations. Some centers experienced challenges using the CT perfusion protocol, leading to the exclusion of several patients from the final analysis due to a lack of interpretable images. We also encountered difficulties in postprocessing CT perfusion data, highlighting an important area of training at implementation. In addition, we observed potential for diagnostic errors in both ancillary investigations and clinical assessments, as well as occasional ambiguity in clinical evaluations, reflecting the real-world complexity of DNC. We used the clinical determination of DNC as the reference standard, which, although widely accepted, has known limitations. DNC is a clinical diagnosis based on the permanent loss of brainstem reflexes and the capacity to breathe, serving as a legally and medically accepted proxy for death. However, it remains only a surrogate marker for the complete cessation of all intracranial neuronal activity. Consequently, imperfections in this reference standard may affect the apparent diagnostic accuracy of ancillary investigations that do not directly measure global neuronal function. Our study therefore compares ancillary investigations with this established, yet imperfect, clinical benchmark rather than with an ideal, but currently unachievable, criterion standard of absolute cessation of all brain functions. Blinded adjudication was performed to reduce uncertainty in reference standards. Nevertheless, some patients were included in the final analysis despite residual ambiguity, which may have contributed to false-positive or false-negative classifications. Most ancillary investigations were performed within the first few days after injury; it remains uncertain whether the accuracy we observed would hold for patients assessed later in their intensive care unit course after prolonged life-sustaining therapies. These issues, however, mirror real-world challenges and ultimately strengthen the credibility and generalizability of our findings. Additionally, clinical and imaging evaluations were performed by experts, an important consideration at implementation at centers without such experience.

## Conclusions

Neither CT perfusion nor CT angiography met the prespecified threshold of greater than 98% for both sensitivity and specificity. Consequently, they should not be used as stand-alone tests to establish DNC. However, they may offer supportive evidence in situations where the bedside examination is incomplete or confounded, but their findings must be weighed against a thorough clinical assessment to minimize false-positive determinations.

## Notes

Supplement 1. **eAppendix 1.** INDex Trial Investigators, Collaborators, and Committees **eAppendix 2.** Methods **eFigure 1.** Subgroup Analyses for Age **eFigure 2.** Subgroup Analyses for Sex **eFigure 3.** Subgroup Analyses for Type of Brain Injury **eFigure 4.** Subgroup Analyses for Artifacts **eTable 1.** Vital Signs and Biologic Parameters at Admission to the Intensive Care Unit **eTable 2.** Clinical Evaluation for Death Determination by Neurologic Criteria – Patient Flow **eTable 3.** Clinical Evaluation for Death Determination by Neurologic Criteria – Results **eTable 4.** Violations – Descriptions **eTable 5.** Clinical Outcomes **eTable 6.** Ancillary Investigation-Related Adverse Events **eTable 7.** Details on False Positive Cases on Qualitative Brainstem CT-Perfusion (n=20) **eTable 8.** Details on False Negative Cases on Qualitative Brainstem CT-Perfusion (n=3) **eTable 9.** Details on False Positive Cases on Qualitative Whole-Brain CT-Perfusion (n=6) **eTable 10.** Details on False Negative Cases on Qualitative Whole-Brain CT-Perfusion (n=13) **eTable 11.** Sensitivity Analysis: Exclusion of Patients Considered Alive Based on Peripheral Movements Alone (n = 279)

Supplement 2. INDex Investigators and Canadian Critical Care Trials Group Members

Supplement 3. Data Sharing Statement
