# Supplementary material for: Computed Tomography Perfusion and Angiography for Death by Neurologic Criteria
Source: JAMA Neurol. 2025 Jun 13;82(9):932–40. doi: 10.1001/jamaneurol.2025.2375 (PMC12166499; doi:10.1001/jamaneurol.2025.2375)
Supplement: Supplement 1. — eAppendix 1. INDex Trial Investigators, Collaborators, and Committees eAppendix 2. Methods eFigure 1. Subgroup Analyses for Age eFigure 2. Subgroup Analyses for Sex eFigure 3. Subgroup Analyses for Type of Brain Injury eFigure 4. Subgroup Analyses for Artifacts eTable 1. Vital Signs and Biologic Parameters at Admission to the Intensive Care Unit eTable 2. Clinical Evaluation for Death Determination by Neurologic Criteria – Patient Flow eTable 3. Clinical Evaluation for Death Determination by Neurologic Criteria – Results eTable 4. Violations – Descriptions eTable 5. Clinical Outcomes eTable 6. Ancillary Investigation-Related Adverse Events eTable 7. Details on False Positive Cases on Qualitative Brainstem CT-Perfusion (n=20) eTable 8. Details on False Negative Cases on Qualitative Brainstem CT-Perfusion (n=3) eTable 9. Details on False Positive Cases on Qualitative Whole-Brain CT-Perfusion (n=6) eTable 10. Details on False Negative Cases on Qualitative Whole-Brain CT-Perfusion (n=13) eTable 11. Sensitivity Analysis: Exclusion of Patients Considered Alive Based on Peripheral Movements Alone (n = 279) [file jamaneurol-e252375-s001.pdf]

Supplemental Online Content

Chassé M, Shankar JJS, Fergusson DA, et al; INDex Investigators and Canadian Critical Care Trials Group. Computed tomography perfusion and angiography for death by neurologic criteria. *JAMA Neurol*. Published online June 13, 2025. doi:10.1001/jamaneurol.2025.2375

eAppendix 1. INDex Trial Investigators, Collaborators, and Committees
eAppendix 2. Methods
eFigure 1. Subgroup Analyses for Age
eFigure 2. Subgroup Analyses for Sex
eFigure 3. Subgroup Analyses for Type of Brain Injury
eFigure 4. Subgroup Analyses for Artifacts
eTable 1. Vital Signs and Biologic Parameters at Admission to the Intensive Care Unit
eTable 2. Clinical Evaluation for Death Determination by Neurologic Criteria – Patient Flow
eTable 3. Clinical Evaluation for Death Determination by Neurologic Criteria – Results
eTable 4. Violations – Descriptions
eTable 5. Clinical Outcomes
eTable 6. Ancillary Investigation-Related Adverse Events
eTable 7. Details on False Positive Cases on Qualitative Brainstem CT-Perfusion (n=20)
eTable 8. Details on False Negative Cases on Qualitative Brainstem CT-Perfusion (n=3)
eTable 9. Details on False Positive Cases on Qualitative Whole-Brain CT-Perfusion (n=6)
eTable 10. Details on False Negative Cases on Qualitative Whole-Brain CT-Perfusion (n=13)
eTable 11. Sensitivity Analysis: Exclusion of Patients Considered Alive Based on Peripheral Movements Alone (n = 279)

This supplemental material has been provided by the authors to give readers additional information about their work.

© 2025 Chassé M et al. *JAMA Neurology*.

eAppendix 1. INDex Trial Investigators, Collaborators, and Committees

Executive Committee

Ian Ball, Michaël Chassé, Sultan Darvesh, Sonny Dhanani, Shane W English, Dean Fergusson, François Lauzier, Jai Jai Shiva Shankar, Alexis F Turgeon

Steering Committee

Ian Ball, Karen Burns, Michaël Chassé, Philippe Couillard, Frederick D'Aragon, Sultan Darvesh, Sonny Dhanani, Shane English, Dean Fergusson, Robert Green, Mathew Hannouche, Salmaan Kanji, Andreas Kramer, François Lauzier, Maureen Meade, Bijoy Menon, Sam Shemie, Jason Shahin, Jai Shankar, Donatella Tampieri, Polina Titova, Alexis Turgeon, Han Ting Wang

Sites

CHU de Montreal (Montreal)

Pierre Aslanian, Sylvain Belisle, François Martin Carrier, Pierre-Marc Chagnon, Annick Chatillon, Daniel Corsilli, Julie Cousineau, Michaël Chassé (PI), Tudor Costacescu, Marc-Jacques Dubois, Andréa Gagnon-Hamelin, Mélissa Gagnon-Hamelin, Martin Girard, François Guilbert, Jean-Gilles Guimond, Christophe Kolan, Dominique Lafrance, Francesca Lamothe, Martine Lebrasseur, Jean-François Lizé, Nicholas Robillard, Catalina Sokoloff (Enrolled 107, Included 88)

McGill University Health Center (Montreal)

Josie Campisi, Jeffrey Chankowsky, Kosar Khwaja, Raham Rahgoshai, Jason Shahin (Site PI) (Enrolled 8, Included 7)

CHU de Québec–Université Laval (Québec City)

David Bellemare, Jean-Luc Gariépy, Stéphanie Grenier, Gabrielle Guilbaut, François Lauzier (Site Co-PI), Alexis Turgeon (Site Co-PI) (Enrolled 22, Included 18)

CHU de Sherbrooke (Sherbrooke)

Marie-Pier Bouchard, Élaine Carbonneau, Jean Chénard, Frederick D'Aragon (Site PI), François Lamontagne, Charles St-Arnaud (Enrolled 29, Included 27)

Montreal Neurological Institute (Montreal)

Erin Cole, Mira Grandillo, Mathew Hannouche (Site PI), Rick Sanchez, Donatella Tampieri, Catherine Therrien, Gilbert Yip (Enrolled 26, Included 23)

Hôpital Maisonneuve-Rosemont (Montreal)

Andrée-Anne Pistono, Danaë Tassy, Han Ting Wang (Site PI) (Enrolled 1, Included 1)

The Ottawa Hospital (Ottawa)

Santanu Chakraborty, Shane English (Site PI), Jessica Haines, Greg Knoll, Doug McGuire, Rebecca Porteous, Irene Watpool (Enrolled 40, Included 36)

St-Michael's Hospital (Toronto)

Andrew Baker (Site PI), Aditya Bharatha, Karen E. A. Burns, Imrana Khalid, Gyan Sandhu, Marlene Santos (Enrolled 10, Included 8)

London Health Sciences (London)

Ian Ball (Site PI), Tracey Bental, Michael Jurkiewicz, Claudio Martin, Marat Slessarev (Enrolled 2, Included 1)

© 2025 Chassé M et al. *JAMA Neurology*.

Hamilton Health Sciences (Hamilton)

Lori Hand, Draga Jichici, Maureen Meade (Site PI), Arun Mensinkai (Enrolled 11, Included 9)

Kingston Health Sciences Centre – Kingston General Hospital (Kingston)

Gordon Boyd (Site PI), Tracy Boyd, Miranda Hunt, Omar Islam (Enrolled 5, Included 0)

William Osler Health Center (Brampton)

Noha Aref, Alexandra Binnie (Site PI), Andrew Gibson, Vinayak Lad, Rosa Myrna Marticorena, Marc Ossip (Enrolled 4, Included 4)

Foothills Medical Center (Calgary)

Cassidy Codan, Philippe Couillard (Site PI), Olesya Dmitrieva, Muneer Eesa, Lily Guan, Andreas Kramer, Julie Kromm (Enrolled 47, Included 40)

Manitoba Health Sciences Centre (Winnipeg)

Khunza Faiz, Nicole Marten, Jai Jai Shiva Shankar (PI), Marco Essig, Maggie Wilson, Ryan Zarychanski (Site PI) (Enrolled 10, Included 10)

QEII Health Sciences Center (Halifax)

Valerie Barette, Mete Erdogan, Robert Green (Site PI), Jane MacLeod, Laura Magennis, Matthias Schmidt, Robert Vandorpe (Enrolled 11, Included 10)

Enrolment Adjudication Committee – Death by neurological criteria

Ian Ball, Michaël Chassé, Sonny Dhananni, Shane English, Dean Fergusson, François Lauzier, Martine Lebrasseur, Polina Titova, Alexis Turgeon

CT-Perfusion and CT-Angiography image interpretation

Marco Essig, Jai Jai Shiva Shankar

Canadian Critical Care Trials Group internal reviewer

Deborah J Cook, Bram Rochwerg

© 2025 Chassé M et al. *JAMA Neurology*.

eAppendix 2. Methods

2.1 DETAILED INCLUSION AND EXCLUSION CRITERIA

Inclusion Criteria

1. Adults ≥ 18 years old
2. Admitted to the intensive care unit for traumatic brain injury, anoxic brain injury, ischemic or hemorrhagic stroke or other causes of brain injury which could induce brain herniation
3. Glasgow Coma Scale (GCS) = 3
4. Sedation stopped for at least 6 hours[a]

Exclusion Criteria

1. Contraindications to CT-perfusion
    1. Pregnancy
    2. Contrast allergy
    3. Kidney injury confirmed by a clinician
    4. Hemodynamic instability that prevents safe transport to the CT-scan
2. Cervical fracture above C6
3. Significant facial trauma limiting cranial nerve examination
4. Hypothermia < 34 °C
5. Use of barbiturates at any time since admission
6. Unresuscitated shock
7. Peripheral nerve or muscle dysfunction or neuromuscular blockade potentially accounting for responsiveness
8. Anoxic brain injury < 72h for patients who underwent therapeutic hypothermia, else the usual 24h waiting period is required as per Canadian national guidelines for death determination
9. Attending physician disagrees to conduct an apnea test or any other abnormalities deemed a confounding factor for death determination by neurologic criteria by the attending clinician

a. We measured plasma concentrations of common sedatives (midazolam, propofol and active metabolites) and analgesics (morphine, hydromorphone, fentanyl and active metabolites) to exclude confounding by these drugs. Details have been published previously[1].

1. Jutras M, Williamson D, Chassé M, Leclair G. Development and validation of a liquid chromatography coupled to tandem mass spectrometry method for the simultaneous quantification of five analgesics and sedatives, and six of their active metabolites in human plasma: Application to a clinical study on the determination of neurological death in the intensive care unit. J Pharm Biomed Anal 2020;190 :113521. DOI: 10.1016/j.jpba.2020.113521.

2.2 PRIMARY OUTCOME DEFINITION

The primary outcome of this study is the diagnostic accuracy (sensitivity and specificity) of brainstem CT-perfusion compared to the reference clinical examination for death by neurologic criteria.

2.3 SECONDARY OUTCOME DEFINITIONS

The secondary outcomes of this study are diagnostic accuracy of whole-brain CT-perfusion (no perfusion anywhere in the brain) and of CT-angiography (10-point, 7-point and 4-point scales), as well as their safety and consistency (inter-rater reliability).

2.4 ANCILLARY INVESTIGATION – CT-PERFUSION AND CT-ANGIOGRAPHY

Once enrolled, all participants underwent a whole-brain CT-perfusion protocol, ensuring coverage of the entire brain. A total of 40 mL of non-ionic iodinated contrast media was injected. Images were acquired according to our previously published imaging protocol, described below. De-identified source images of CT perfusion were sent from each of the study centres to a central imaging core lab to be processed and analyzed. The CT perfusion images were post processed using a vendor neutral post-processing software (Olea Sphere 3.0, Olea Medical, Cambridge, USA) to standardize the post-processing technique. CT-perfusion results were interpreted and recorded independently by two neuroradiologists blinded from each other and to the clinical history. Imaging interpretation disagreements were resolved by consensus using a third reading, blinded from the first two interpretations and the reference standard.

CT-perfusion interpretation

For quantitative brainstem CT-perfusion, a computer assessed large regions of interest on axial slices at the medulla, pons and midbrain.

For qualitative CT-perfusion interpretation, images were assessed for a matched decrease in cerebral blow flow and cerebral blood volume.

Definition of death by neurologic criteria – brainstem CT-perfusion

For quantitative brainstem CT-perfusion, the test was judged positive for DNC if cerebral blood flow was below 10mL/100g/min and if cerebral blood volume was below 2mL/100g on at least two consecutive 5 mm axial slices of brainstem.

For qualitative brainstem CT-perfusion, the test was judged positive for DNC when there was a qualitative matched decrease in cerebral blood flow and volume in the brainstem, regardless of findings in the rest of the brain.

The CT-perfusion result was considered a true positive or a true negative when CT-perfusion agreed with the result of the reference standard. The CT-perfusion result was considered a false positive or a false negative when in disagreement with the reference standard.

Definition of death by neurologic criteria – whole-brain CT-perfusion

For whole-brain CT-perfusion, the test was considered positive for DNC when there was a qualitative matched decrease in cerebral blood flow and volume in the entire brain.

© 2025 Chassé M et al. *JAMA Neurology*.

CT-angiography interpretation

For CT-angiography, neuroradiologists assessed the M4 segments of the middle cerebral arteries, the A3 segments of the anterior cerebral arteries, the P2 segments of the posterior cerebral arteries, the basilar artery, the internal cerebral veins and the great vein of Galen for vessel opacification.

Definition of death by neurologic criteria – CT-angiography

CT-angiographies were considered positive for DNC based on three standardized scales (10-point, 7-point and 4-point scales), each applied separately to the early and late acquisition phases:

4-point scale: no opacification in the internal cerebral veins or M4 segments of the middle cerebral arteries.

7-point scale: no opacification in the internal cerebral veins, the great vein of Galen, the M4 segments of the middle cerebral arteries or the A3 segments of the anterior cerebral arteries.

10-point scale: no opacification in the internal cerebral veins, the great vein of Galen, the M4 segments of the middle cerebral arteries, the A3 segments of the anterior cerebral arteries, the P2 segments of the posterior cerebral arteries or the basilar artery.

The CT-angiography result was considered a true positive or a true negative when CT-angiography agreed with the result of the reference standard. The CT-angiography result was considered a false positive or a false negative when in disagreement with the reference standard.

2.5 Ancillary Investigation Imaging Protocol

Minimum requirement for the CT scanner

- At least 64 slice scanner
- Had a CT perfusion acquisition protocol (or was programmed for CTP image acquisition).
- CT perfusion protocol covered at least 8 cm coverage of the brain in z-axis

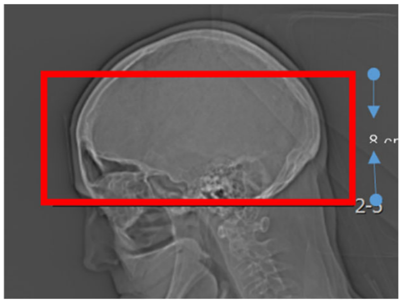

Images sent to the core lab had the following:
• **Plain Head CT**: Axial images of whole head with a slice thickness of 5 mm in brain window.<br>• **CT-Perfusion**: Axial images with a slice thickness of 5mm without overlap.<br>• **CT-Angiography**: Axial images from the peak arterial phase, with a section thickness of 1.5 mm.

© 2025 Chassé M et al. *JAMA Neurology*.

CTP Protocol - Acquisition parameters

- 80 kV, 100 mAs
- Minimum of 8-cm-coverage in z-axis- either with static table or 'shuttle mode'. ***Most important:*** The volume of brain covered included foramen magnum as the inferior-most slice (see the image) - for complete coverage of the brainstem.
- **Time of acquisition:** at least 60 seconds with optimal temporal resolution of every 2 seconds in in-flow phase and every 5 seconds in the out-flow phase.
- **Contrast injection protocol:** Contrast was injected through a 18 G (minimum size) IV cannula in the branchial vein (or proximal vein or femoral vein). A total of 40 mL of nonionic iodinated contrast media (equivalent to Isovue 370) was injected at a rate of 5 mL/s, followed by a saline flush of 40-mL sodium chloride at 5 mL/s and a start delay of 5 seconds.
    - ***NOTE-*** *In case the IV contrast injection went interstitial, the injection was immediately stopped and the vein was reassessed. Repeat CTP could be acquired at the same sitting with contrast injection through a different vein, preferably in a different extremity.*
- **Images (in DICOM format) were sent:** Axial source images with a slice thickness of **5 mm** without overlap for CT perfusion analysis.
- **NO PERFUSION POST-PROCESSING WAS DONE.** CTP was only processed at the imaging core lab.
- **CTP was not reported locally prior to clinical evaluation and consensus.**

For CT-angiography Analysis

- **Whole CTP raw data in 1.5 mm thick slices were sent**

OR

- Using the time-density curve, the phase at the peak arterial phase (see the red arrow in the following image) was selected.
- In case the raw data was not 1.5 mm thick, axial images were reconstructed from the peak arterial phase as well as from the last phase (green arrow in the following image) without overlap with a section thickness of 1.5 mm. This was sent in DICOM format.

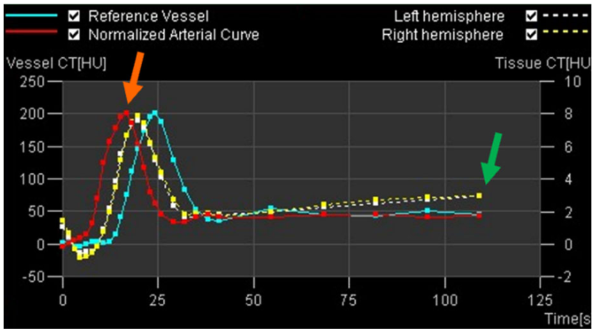

© 2025 Chassé M et al. *JAMA Neurology*.

2.6 ANCILLARY INVESTIGATION CASE REPORT FORM

**1. Plain CT Head**: complete this section if Yes checked for CT Head	
1.1. Diffuse loss of Grey-white differentiation compatible with diffuse cerebral edema	□ Yes □ No
1.2. Remarks	

**2. CT-Perfusion (CTP)**: complete this section if Yes checked for CTP	
2.1. Contrast opacification in extracranial vessels	□ Appearance □ Disappearance
2.2. Whole brain matched cerebral blood flow (CBF) & cerebral blood volume (CBV) defect	□ Yes □ No
2.3. Brainstem matched CBF & CBV defect	□ Yes □ No
2.4. Isolated Brainstem matched CBF & CBV defect	□ Yes □ No
2.5. CBF in brainstem (in ml/100 gm/min)	____________
2.6. CBV in brainstem (in ml/100 gm)	____________
2.7. Remarks	

**3. CT-Angiogram (CTA)**: Complete this section if Yes checked for CTA			
3.1. CTA contrast opacification in **peak phase**		Right	Left
	Extracranial	□ Yes □ No	□ Yes □ No
	Supraclinoid Internal Carotid Artery (ICA)	□ Yes □ No	□ Yes □ No
	Middle Cerebral Artery (MCA) (M4 or cortical segments)	□ Yes □ No	□ Yes □ No
	Anterior Cerebral Artery (ACA) (A2/3 segment)	□ Yes □ No	□ Yes □ No
	Posterior Cerebral Artery (PCA) (P2 segment)	□ Yes □ No	□ Yes □ No
	Intracranial Vertebral Artery	□ Yes □ No	□ Yes □ No
	Basilar Artery (BA)	□ Yes □ No	□ Yes □ No
	Internal Cerebral Vein (ICV)	□ Yes □ No	□ Yes □ No

© 2025 Chassé M et al. *JAMA Neurology*.

	Vein of Galen (VOG)	□ Yes □ No
3.2. Death by neurologic criteria on CTA	4-point scale (MCA & ICV)	□ Yes □ No
	7-point scale (MCA, ACA, ICV & VOG)	□ Yes □ No
	10-point scale (MCA, ACA, PCA, BA, ICV & VOG)	□ Yes □ No
3.3. Remarks		

**4. CT-Angiogram (CTA)**: Complete this section if Yes checked for CTA			
4.1. CTA contrast opacification in **<u>late phase</u>**		Right	Left
	Extracranial	□ Yes □ No	□ Yes □ No
	Supraclinoid Internal Carotid Artery (ICA)	□ Yes □ No	□ Yes □ No
	Middle Cerebral Artery (MCA) (M4 or cortical segments)	□ Yes □ No	□ Yes □ No
	Anterior Cerebral Artery (ACA) (A2/3 segment)	□ Yes □ No	□ Yes □ No
	Posterior Cerebral Artery (PCA) (P2 segment)	□ Yes □ No	□ Yes □ No
	Intracranial Vertebral Artery	□ Yes □ No	□ Yes □ No
	Basilar Artery (BA)	□ Yes □ No	□ Yes □ No
	Internal Cerebral Vein (ICV)	□ Yes □ No	□ Yes □ No
	Vein of Galen (VOG)	□ Yes □ No	
4.2. Death by neurologic criteria on CTA	4-point scale (MCA & ICV)	□ Yes □ No	
	7-point scale (MCA, ACA, ICV & VOG)	□ Yes □ No	
	10-point scale (MCA, ACA, PCA, BA, ICV & VOG)	□ Yes □ No	
4.3. Remarks			

**5. Additional Ancillary Imaging Tests**	
5.1. Any other imaging modality used:	□ Yes □ No<br>If Yes, please specify:<br>________________________________
5.2. Remarks	

© 2025 Chassé M et al. *JAMA Neurology*.

2.7 Reference Standard – Clinical Examination for Death by Neurologic Criteria

Shortly after completion of the CT-perfusion (to enhance validity, the shorter the delay, the better), two staff neurologists, neurosurgeons or intensivists blinded to any imaging results performed and recorded a complete clinical death by neurologic criteria assessment using standardized criteria based on contemporary Canadian guidelines. There was minimal time delay between CT perfusion and clinical examination (<2 hours) to enhance validity of CT perfusion (the shorter the delay, the better).

Definition of death by neurologic criteria – reference standard

A patient was considered deceased in the presence of all of the following:

1) an established etiology capable of causing death by neurological criteria;
2) an absence of confounders that can mimic death by neurologic criteria;
3) an absence of all brainstem reflexes; and
4) a positive apnea test.

The two clinical examiners then discussed their clinical neurological evaluation and classified patients as clinically neurologically deceased or not. Disagreements were resolved by consensus at the bedside. If a consensus could not be reached by two clinical reviewers, a third clinical reviewer was to be invited to conduct an additional neurological evaluation and try to reach consensus with the two other clinicians. This did not occur during the study.

The clinical evaluation was considered the reference standard of death by neurologic criteria for the study.

2.8 Reference Standard Case Report Form

**1. EXAMINER #1**		Date: ___ ___ / ___ ___ ___ / 20___ ___<br>Time (24hr): __ __ : __ __	
Bilateral visualization of eardrums		□ Yes<br>□ No if no, reason: ________________	
**Hemodynamic status**			
At the time of the exam		BP _____/______ (mmHg)	
		HR _____	
		T _______ °C<br>□ Rectal □ Oral □ Axillary<br>□ Other, specify ________	
**Glasgow Coma Scale 3/15**		Eyes: _____<br>Verbal: 1 (T)<br>Motor: _____<br>Total: _____	
**Neurological exam**		Cranial nerve territory response	Peripheral nerve territory response
Painful stimulation	Frontal	□ Absent □ Present	□ Absent □ Present
	Maxillary	□ Absent □ Present	□ Absent □ Present
	Trapezius	□ Absent □ Present	□ Absent □ Present
	Lower Limbs	□ Absent □ Present	□ Absent □ Present
	Upper Limbs	□ Absent □ Present	□ Absent □ Present

© 2025 Chassé M et al. *JAMA Neurology*.

If presence of a peripheral response, is that response a spinal reflex? **(complete peripheral response form)**		□ Yes, it is a spinal reflex<br>□ Both spinal and upper motoneuron mediated reflexes present<br>□ No, it is an upper motoneuron mediated reflex<br>□ Unsure
Pupillary response to light	Right	□ Absent □ Present
	Left	□ Absent □ Present
Corneal response	Right	□ Absent □ Present
	Left	□ Absent □ Present
Oculo-cephalic response (Doll's eyes)	Right	□ Absent □ Present
	Left	□ Absent □ Present
Vestibulo-ocular response (oculo-caloric)	Right	□ Absent □ Present
	Left	□ Absent □ Present
	If present, please describe:	____
Cough reflex		□ Absent □ Present
Pharyngeal (gag) reflex		□ Absent □ Present
Is an apnea test required to confirm death?<br>(An apnea test is optional if presence of any other brainstem reflex above)		□ Yes □ No
**Apnea test** (if applicable)	Apnea test procedure (check all that apply)	□ Connected to ventilator*<br>o Peep: ________________<br>o Mode: ________________<br>o Flow: ________________<br>o I:E Ratio: ________________<br>o Level of assistance: ___________________<br>□ T-tube with Peep valve, value: ______________________<br>□ T-tube without Peep valve<br>□ Intra tracheal O2 canula<br>□ Intra tracheal CO2 canula<br>* If presence of respiratory efforts while connected to ventilator, test has to be repeated with T-tube to exclude auto-triggering

	PH	PaO_2	$PaCO_2$	SaO_2
At 0 min (time: ________):				
At end of test (time: ______):				

Test stopped at:	_____ min Reason: ______________________
Respiratory effort	□ Yes □ No
Neurological evaluation conducted by:	□ Neurologist<br>□ Neurosurgeon<br>□ Intensivist<br>□ Resident (Last year of specialty training)<br>o Specify resident specialty: ________________<br>□ Other, specify: ____________________________

© 2025 Chassé M et al. *JAMA Neurology*.

Were you aware of the study CTP or CTA result prior to this clinical evaluation?	□ Yes □ No<br>If Yes, explain: ______________________________<br>____<br>____
Were your aware of any ancillary imaging test performed for death determination by neurologic criteria prior to this clinical evaluation?	□ Yes □ No<br>If Yes, explain: ______________________________<br>____<br>____
**Is the clinical evaluation compatible with death by neurologic criteria?**<br><br>**1) Brain injury sufficient to cause death by neurologic criteria;**<br>**2) Absence of confounding factor that can mimic death by neurologic criteria;**<br>**3) Absence of brainstem reflexes;**<br>**4) Positive apnea test.**	□ Yes □ No<br><br>Date: ___ ___ / ___ ___ ___ / 20___ ___<br><br>Time (24hr): __ __ : __ __

**1. Reconciliation Form (After completing Exam 1 & Exam 2):**	
**Final diagnosis**	□ Death by neurologic criteria declared<br>□ Death by neurologic criteria **not** declared<br>if no, reason: __________________________<br>______________________________________<br>______________________________________<br>______________________________________<br>______________________________________<br>______________________________________<br>______________________________________<br><br>□ Examiners can't agree on a final diagnosis<br>Reason:________________________________<br>______________________________________<br>______________________________________<br>______________________________________<br>______________________________________<br>______________________________________<br>______________________________________

© 2025 Chassé M et al. *JAMA Neurology*.

2.9 Adverse Events Definitions

Expected Adverse Events were pre-defined as follows:

A. **Hypertension:** more than 180 mmHg systolic for 2 minutes.
B. **Hypotension:** less than 60 mmHg mean arterial pressure for 2 minutes.
C. **New desaturation:** SpO2 less than 88% for more than 1 minute.
D. **Accidental extubation*:** unanticipated removal of the endotracheal tube.
E. **New catheter dysfunction:** obstruction or removal of an IV catheter.
F. **Code blue***: cardiac arrest or pre-cardiac arrest.
G. **Other**
H. **Iodine injection related events**
   a. **Minor adverse events (self-limiting, non-progressive):** New limited urticaria, limited cutaneous edema, rhinorrhea, or conjunctivitis.
   b. **Moderate adverse events (often require management):** New diffuse urticaria, facial edema, bronchospasm, or mild hypoxia.
   c. **Severe reactions (often require intervention)*:** New diffuse erythema/edema with hypotension, laryngeal edema with strider and/or hypoxia, wheezing/bronchospasm, significant hypoxia, or anaphylactic shock (hypotension + tachycardia).

Events marked by an * had to be reported to coordinating center within 24h

2.10 Adjudication of Death by Neurological Criteria

Study documentation for determination of death by neurologic criteria (DNC) was reviewed by an independent adjudication committee composed of intensivists with expertise in neurocritical care and DNC (Supplementary Material Section 1). Blinded to CT-perfusion results, the committee assessed eligibility of all enrolled patients based on pre-specified criteria (Supplementary Material Section 2). Only patients with complete clinical evaluations deemed unconfounded by the committee were included in the final analyses. In rare instances, marked with an asterisk (*) in Table S3, the committee identified discrepancies in the reported clinical evaluations that should have technically disqualified DNC determination by clinical examination alone. However, because the patients in these cases were ultimately declared DNC by two trained clinicians without any identifiable confounding factors, after following all other study procedures, they remained included in the analyses.

2.11 Timing of the Analyses and Statement on the Statistical Analytic Plan

The study was registered with ClinicalTrials.gov (NCT03098511) on March 27, 2017, prior to the enrollment of the first patient. The study protocol, including the pre-specified statistical analysis plan, received initial Research Ethics Board (REB) approval on April 5, 2017, and remained unchanged throughout the study. In August 2020, to account for a higher-than-anticipated rate of protocol non-completion (detailed in Figure 1), the executive committee amended the protocol to increase the target enrollment to 330 patients. This adjustment aimed to ensure that the final number of patients completing the study protocol and included in the primary analysis would remain at or above the originally planned 270. This amendment received REB approval on August 25, 2020.

© 2025 Chassé M et al. *JAMA Neurology*.

eFigure 1. Subgroup Analyses for Age

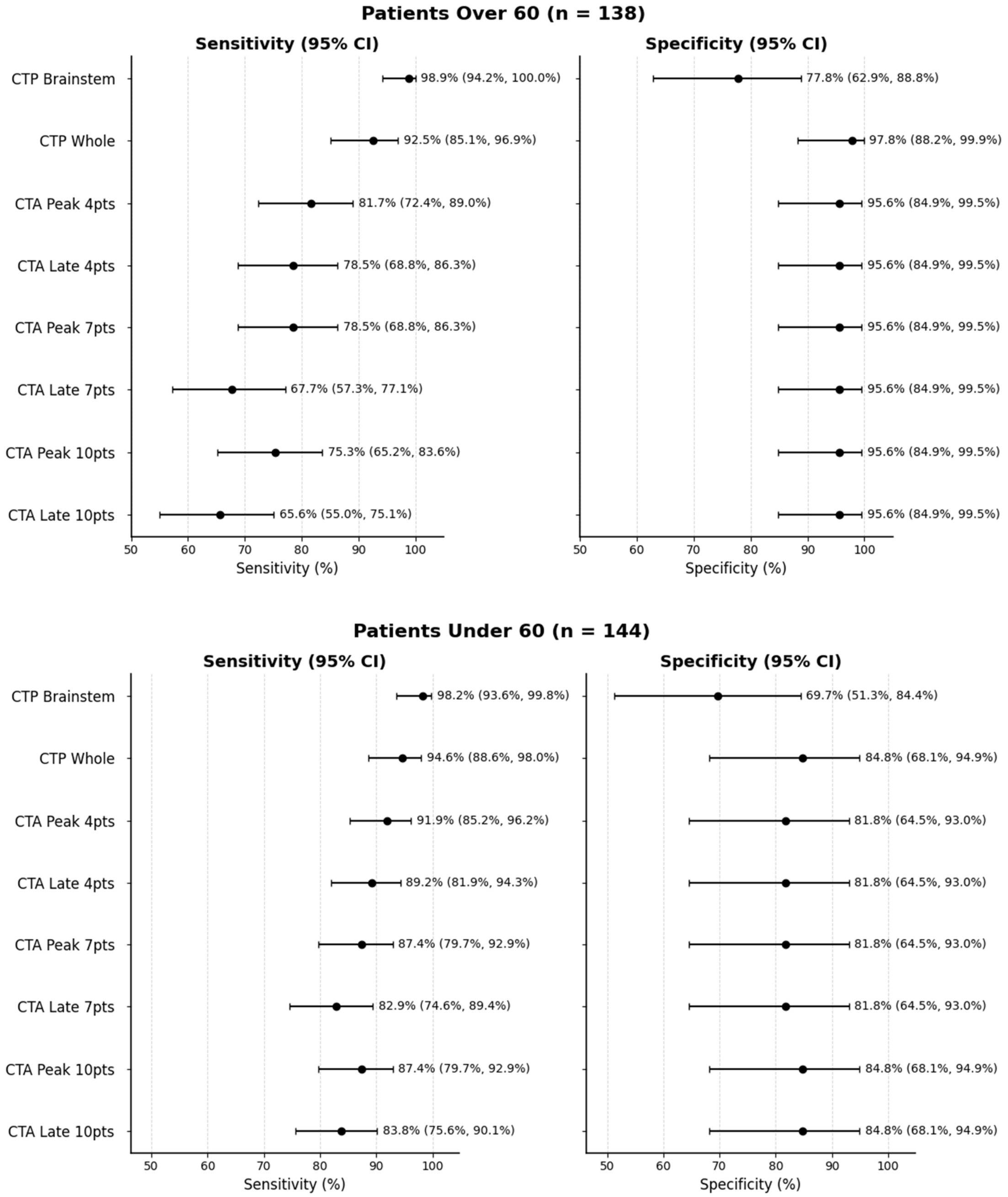

© 2025 Chassé M et al. *JAMA Neurology*.

eFigure 2. Subgroup Analyses for Sex

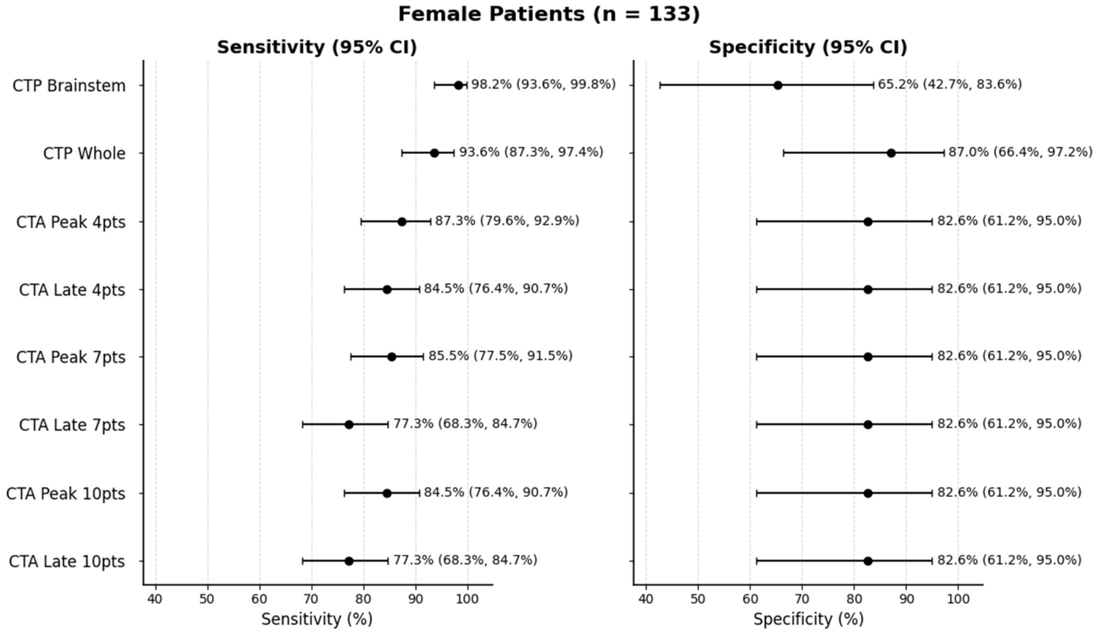

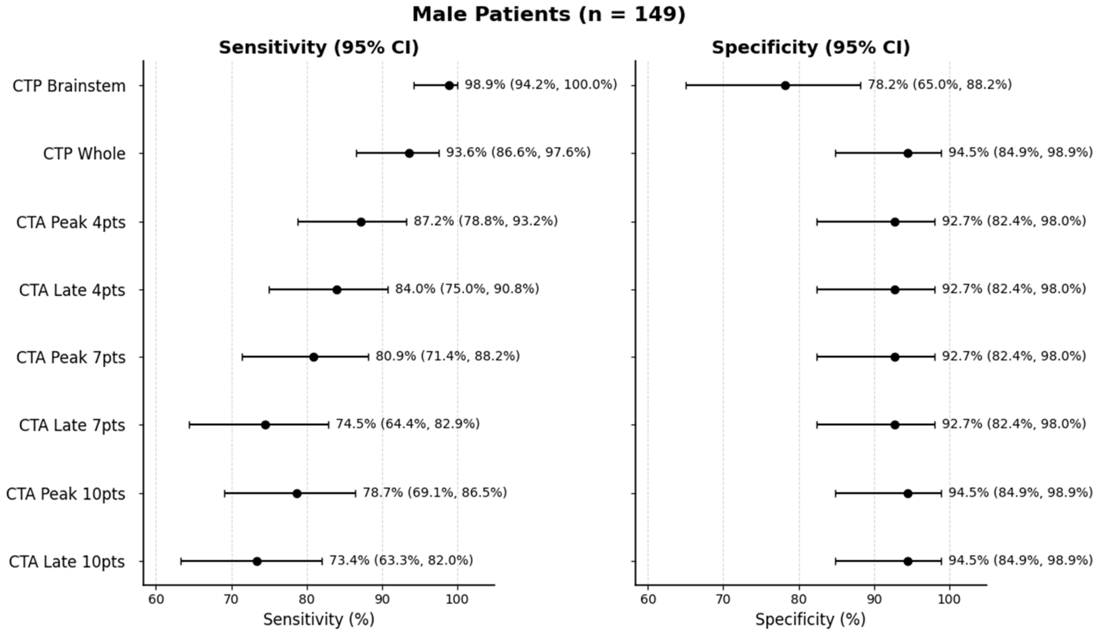

© 2025 Chassé M et al. *JAMA Neurology*.

eFigure 3. Subgroup Analyses for Type of Brain Injury

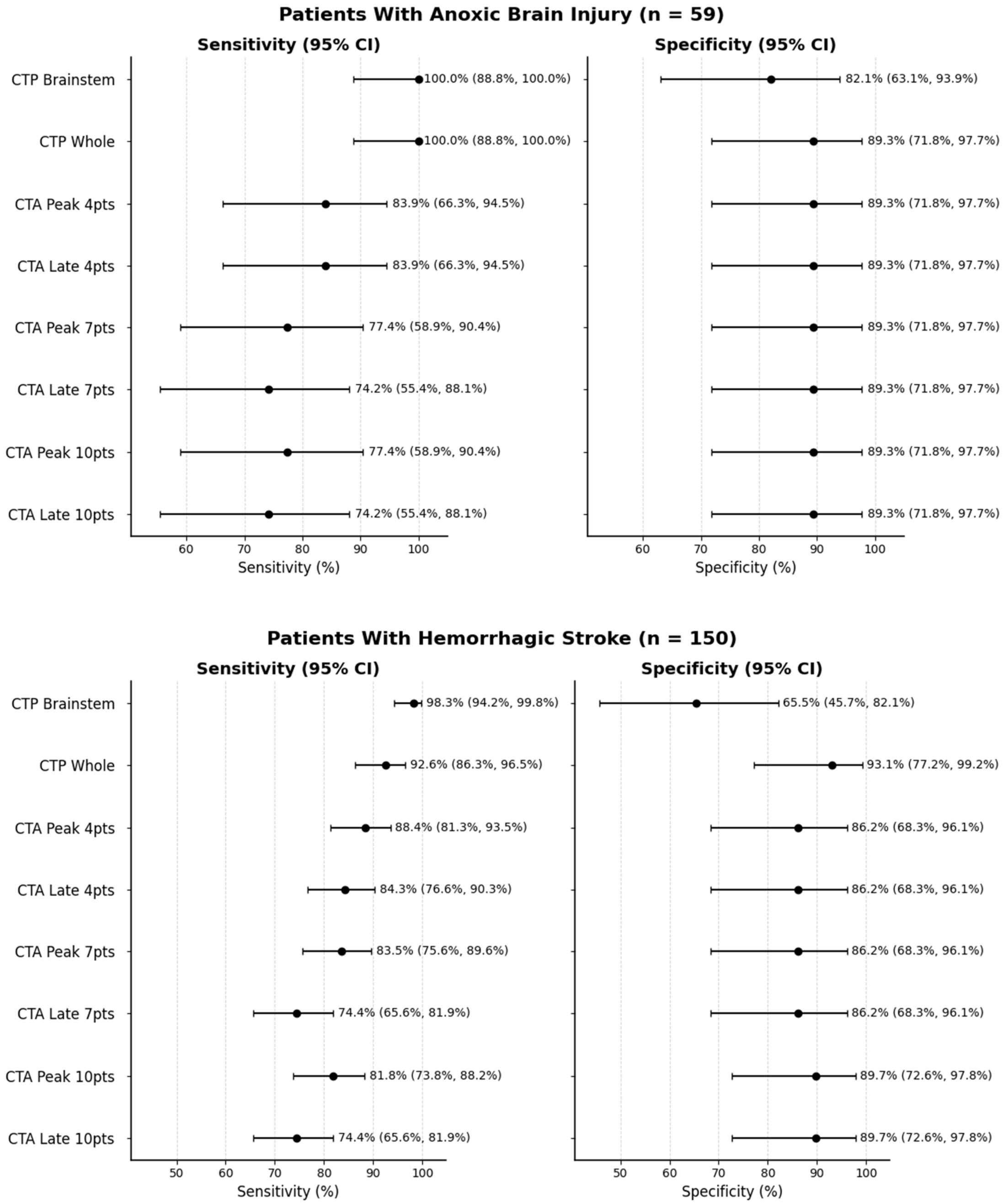

© 2025 Chassé M et al. *JAMA Neurology*.

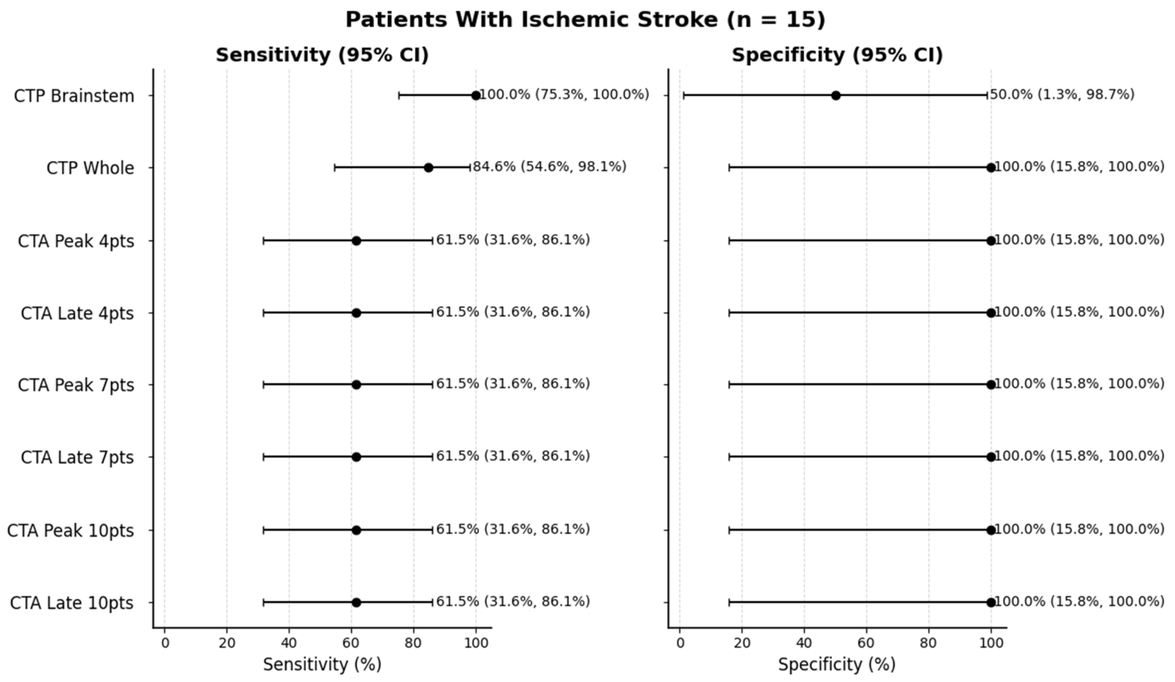

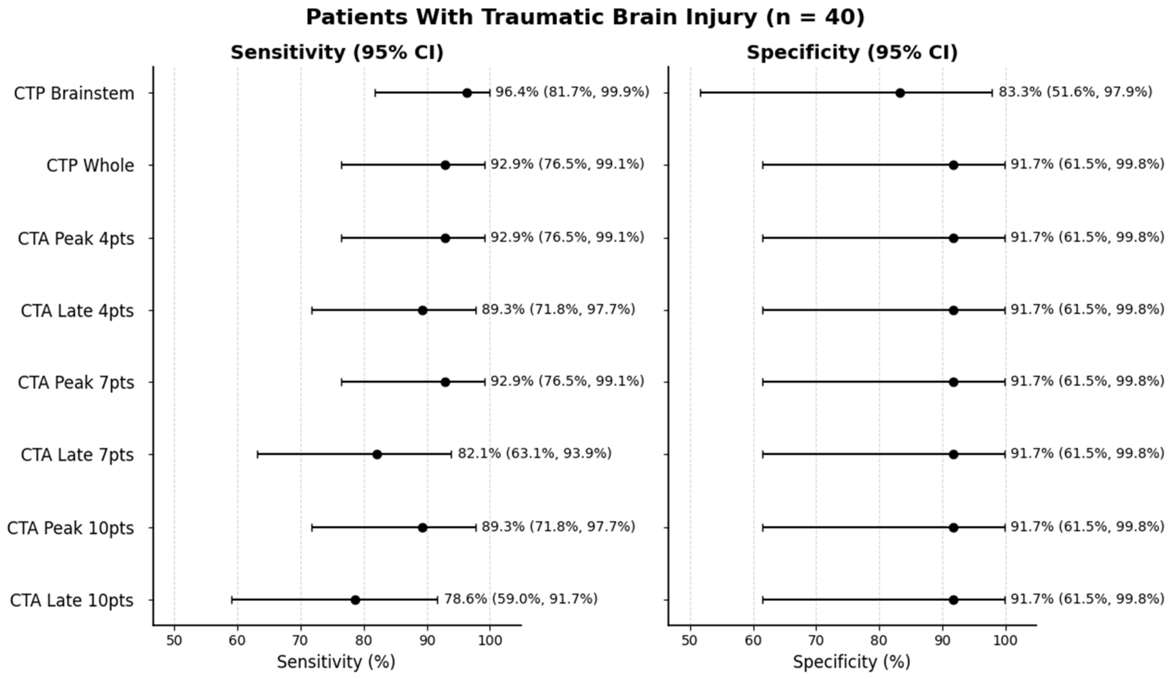

© 2025 Chassé M et al. *JAMA Neurology*.

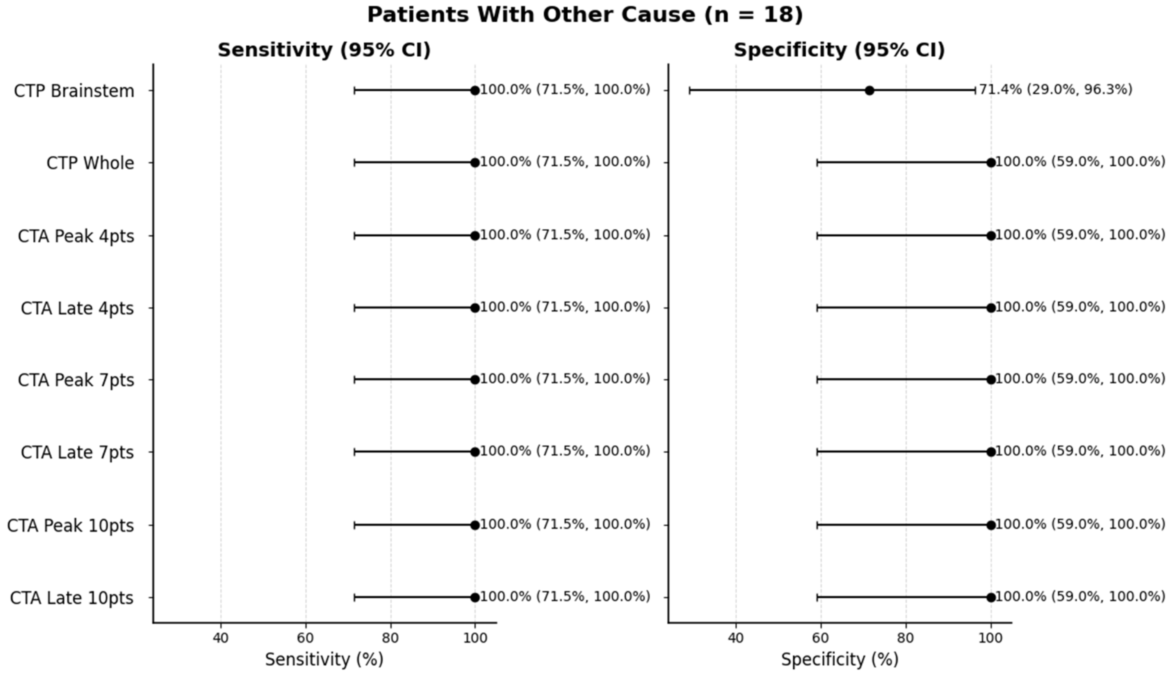

© 2025 Chassé M et al. *JAMA Neurology*.

eFigure 4. Subgroup Analyses for Artifacts

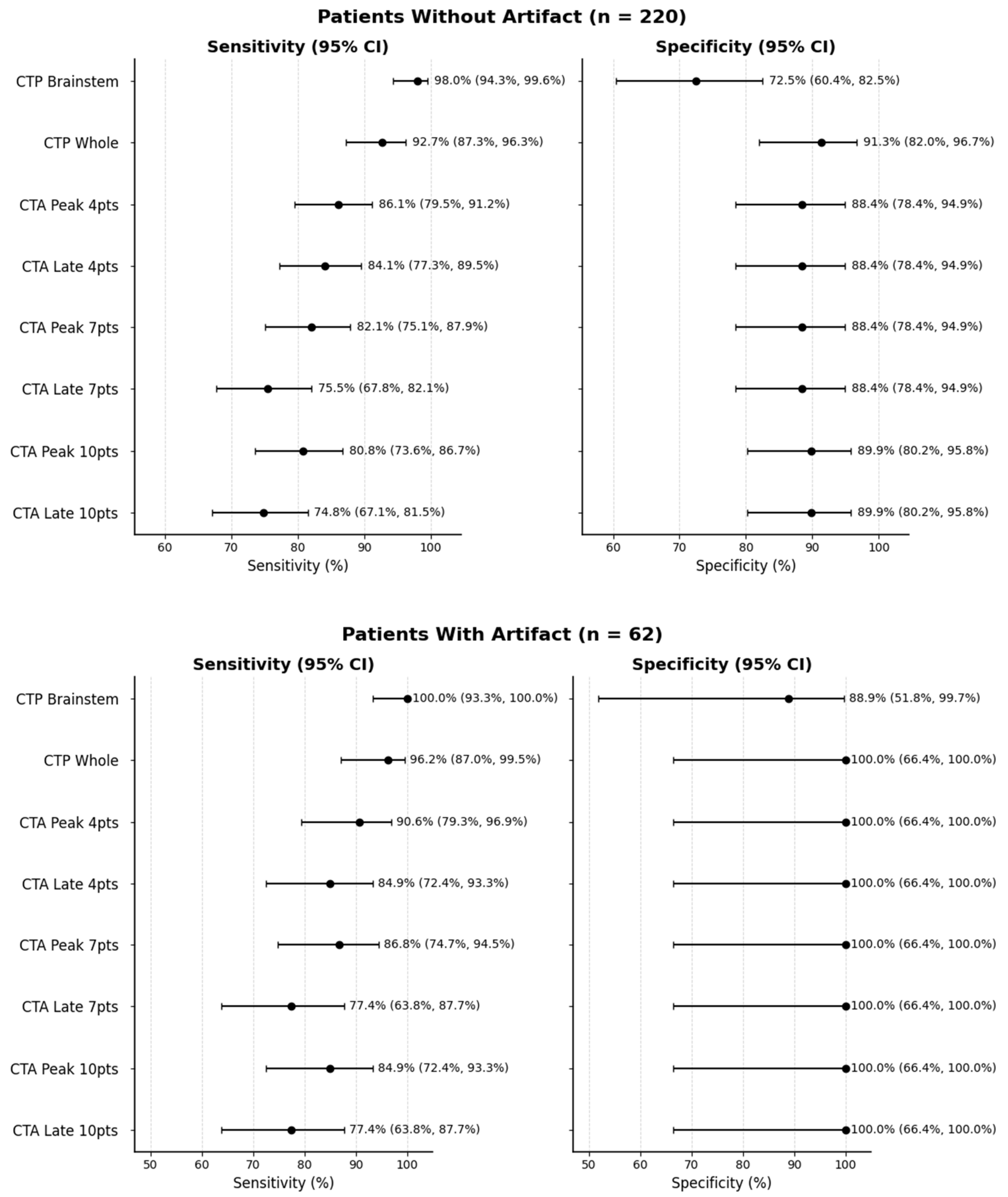

© 2025 Chassé M et al. *JAMA Neurology*.

eTable 1. Vital Signs and Biologic Parameters at Admission to the Intensive Care Unit

	**Entire cohort N=282**	**Deceased n=204**	**Alive n=78**
Vital signs (mean, SD)			
Systolic blood pressure (mmHg)	137.6 (33.7)	137.1 (35.8)	138.9 (27.4)
Diastolic blood pressure (mmHg)	76.3 (19.8)	76.6 (20.5)	75.6 (18.0)
Mean arterial pressure (mmHg)	97.3 (23.9)	97.2 (24.9)	97.7 (21.2)
Heart rate (bpm)	86.3 (23.1)	86.7 (24.6)	85.3 (18.7)
Body temperature (°C)	35.8 (1.5)	35.7 (1.5)	36.0 (1.3)
Biologic parameter (mean, SD)			
Blood glucose (mmol/L)	10.6 (4.4)	10.4 (4.5)	11.0 (4.1)
Hemoglobin (g/L)	123.6 (22.4)	122.5 (22.5)	126.4 (21.9)
Sodium (mmol/L)	140.4 (6.1)	140.6 (6.4)	139.8 (5.3)
Potassium (mmol/L)	3.9 (0.7)	3.9 (0.7)	3.9 (0.7)
Creatinine (μmol/L)	90.4 (95.3)	86.1 (80.6)	101.6 (125.7)
Urea (mmol/L)	6.3 (4.6)	6.3 (4.9)	6.6 (3.6)
pH	7.4 (0.1)	7.4 (0.1)	7.3 (0.1)
Bicarbonate (HCO_3, mmol/L)	21.3 (4.5)	21.5 (4.2)	20.8 (5.3)
Arterial partial pressure of CO_2 ($PaCO_2$; mmHg)	37.9 (9.9)	37.6 (9.5)	38.7 (11.0)
Arterial partial pressure of O_2 (PaO_2; mmHg)	147.2 (87.4)	148.3 (87.1)	144.1 (88.8)
Fraction of inspired O_2 (FiO_2; %)	52.2 (23.2)	52.0 (22.7)	52.9 (24.6)

© 2025 Chassé M et al. *JAMA Neurology*.

eTable 2. Clinical Evaluation for Death Determination by Neurologic Criteria – Patient Flow

	Entire cohort N=282	Deceased n=204	Alive n=78
Patient flow (median, IQR)			
Time (mins) between ICU departure and ancillary investigation	15.0 (11.0 – 22.0)	15.0 (11.0 – 23.0)	14.0 (10.0 – 19.8)
Time (mins) between ancillary investigation and return to ICU	15.0 (10.0 – 21.0)	16.5 (11.0 – 22.0)	12.5 (9.0 – 18.8)
Time (mins) between ICU departure and return to ICU	32.0 (24.0 – 40.8)	34.0 (25.0 – 42.2)	28.5 (22.2 – 35.0)
Time (mins) between DNC assessment and return to ICU	44.5 (22.0 – 71.0)	48.0 (29.0 – 75.2)	28.0 (15.0 – 59.0)

© 2025 Chassé M et al. *JAMA Neurology*.

eTable 3. Clinical Evaluation for Death Determination by Neurologic Criteria – Results

	Examiner 1			Examiner 2		
**Assessment**	**Entire cohort N=282**	**Deceased n=204**	**Alive n=78**	**Entire cohort N=282**	**Deceased n=204**	**Alive n=78**
Pre-requisites						
Bilateral visualization of eardrums (n, %)	257 (91.1%)	187 (91.7%)	70 (89.7%)	259 (91.8%)	188 (92.2%)	71 (91.0%)
Systolic blood pressure (mmHg; mean, SD)	134.7 (25.2)	129.6 (23.0)	147.8 (26.3)	135.2 (25.1)	130.4 (23.2)	147.8 (25.6)
Diastolic blood pressure (mmHg; mean, SD)	70.1 (13.8)	70.5 (13.9)	69.0 (13.8)	70.8 (15.2)	71.1 (16.1)	70.0 (12.8)
Heart rate (bpm; mean, SD)	84.0 (18.9)	82.1 (18.5)	88.9 (19.4)	84.5 (20.1)	82.5 (19.0)	89.9 (21.9)
Temperature (°C; mean, SD)	36.4 (1.0)	36.1 (0.9)	37.2 (1.0)	36.4 (1.0)	36.1 (0.8)	37.2 (1.0)
Glasgow Coma Scale (median, IQR)						
GCS eyes	1 (1 – 1)	1 (1 – 1)	1 (1 – 1)	1 (1 – 1)	1 (1 – 1)	1 (1 – 1)
GCS motor	1 (1 – 1)	1 (1 – 1)	1 (1 – 1)	1 (1 – 1)	1 (1 – 1)	1 (1 – 1)
GCS total	3 (3 – 3)	3 (3 – 3)	3 (3 – 3)	3 (3 – 3)	3 (3 – 3)	3 (3 – 3)
Motor responses (n, %)						
Facial motor response to supra-orbital painful stimulus	5 (1.8%)	0 (0.0%)	5 (6.4%)	5 (1.8%)	0 (0.0%)	5 (6.4%)
Peripheral motor response to supra-orbital painful stimulus	21 (7.4%)	1 (0.5%)[a]	20 (25.6%)	21 (7.4%)	1 (0.5%) [a]	20 (25.6%)
Facial motor response to maxillary painful stimulus	5 (1.8%)	0 (0.0%)	5 (6.4%)	5 (1.8%)	0 (0.0%)	5 (6.4%)
Peripheral motor response to maxillary painful stimulus	22 (7.8%)	1 (0.5%) [a]	21 (26.9%)	22 (7.8%)	1 (0.5%) [a]	21 (26.9%)
Facial motor response to trapezius painful stimulus	9 (3.2%)	0 (0.0%)	9 (11.5%)	8 (2.8%)	0 (0.0%)	8 (10.3%)
Peripheral motor response to trapezius painful stimulus	25 (8.9%)	0 (0.0%)	25 (32.1%)	27 (9.6%)	0 (0.0%)	27 (34.6%)
Motor response in lower limbs to painful stimulus applied to cranial nerve territory	13 (4.6%)	6 (2.9%) [a]	7 (9.0%)	10 (3.5%)	2 (1.0%) [a]	8 (10.3%)
Motor response in lower limbs to painful stimulus applied to peripheral nerve territory	101 (35.8%)	60 (29.4%)	41 (52.6%)	101 (35.8%)	57 (27.9%)	44 (56.4%)
Motor response in upper limbs to painful stimulus applied to cranial nerve territory	7 (2.5%)	1 (0.5%) [a]	6 (7.7%)	8 (2.8%)	1 (0.5%) [a]	7 (9.0%)

© 2025 Chassé M et al. *JAMA Neurology*.

Motor response in upper limbs to painful stimulus applied to peripheral nerve territory	38 (13.5%)	10 (4.9%)	28 (35.9%)	42 (14.9%)	11 (5.4%)	31 (39.7%)
Interpretation of motor response (n, %)						
Spinal reflex	82 (29.1%)	64 (31.4%)	18 (23.1%)	82 (29.1%)	63 (30.9%)	19 (24.4%)
Upper motor neuron mediated response	15 (5.3%)	0 (0%)	15 (19.2%)	16 (5.7%)	0 (0.0%)	16 (20.5%)
Both spinal reflex and upper motor neuron mediated response	12 (4.3%)	0 (0%)	12 (15.4%)	13 (4.6%)	0 (0.0%)	13 (16.7%)
Uncertain	11 (3.9%)	2 (1.0%) [a]	9 (11.5%)	10 (3.5%)	2 (1.0%)[a]	8 (10.3%)
Cranial nerve responses (n, %)						
Pupillary response to light (right eye)	34 (12.1%)	0 (0.0%)	34 (43.6%)	31 (11.0%)	0 (0.0%)	31 (39.7%)
Pupillary response to light (left eye)	32 (11.3%)	0 (0.0%)	32 (41.0%)	30 (10.6%)	0 (0.0%)	30 (38.5%)
Corneal response (right eye)	35 (12.4%)	0 (0.0%)	35 (44.9%)	35 (12.4%)	0 (0.0%)	35 (44.9%)
Corneal response (left eye)	33 (11.7%)	0 (0.0%)	33 (42.3%)	33 (11.7%)	0 (0.0%)	33 (42.3%)
Oculo−cephalic response (right eye)	21 (7.4%)	0 (0.0%)	21 (26.9%)	21 (7.4%)	0 (0.0%)	21 (26.9%)
Oculo−cephalic response (left eye)	21 (7.4%)	0 (0.0%)	21 (26.9%)	20 (7.1%)	0 (0.0%)	20 (25.6%)
Caloric vestibulo-ocular response (right eye)	20 (7.1%)	0 (0.0%)	20 (25.6%)	21 (7.4%)	0 (0.0%)	21 (26.9%)
Caloric vestibulo−ocular response (left eye)	21 (7.4%)	0 (0.0%)	21 (26.9%)	20 (7.1%)	0 (0.0%)	20 (25.6%)
Cough reflex	53 (18.8%)	0 (0.0%)	53 (67.9%)	52 (18.4%)	0 (0.0%)	52 (66.7%)
Pharyngeal (gag) reflex	30 (10.6%)	0 (0.0%)	30 (38.5%)	26 (9.2%)	0 (0.0%)	26 (33.3%)
Apnea test						
pH at test start (mean, SD)	7.4 (0.1)	7.4 (0.1)	7.4 (0.1)	7.4 (0.1)	7.4 (0.1)	7.4 (0.1)
Arterial partial pressure of O_2 (PaO_2) at test start (mmHg; mean, SD)	281.6 (139.9)	282.0 (138.7)	276.2 (161.0)	280.6 (138.1)	281.6 (136.7)	267.3 (159.5)
Arterial partial pressure of CO_2 ($PaCO_2$) at test start (mmHg; mean, SD)	39.7 (5.4)	39.6 (5.3)	40.7 (6.8)	39.9 (5.8)	39.7 (5.3)	42.6 (10.0)
Arterial saturation of O_2 (SaO_2) at test start (%; mean, SD)	97.8 (8.7)	97.8 (9.0)	98.3 (1.7)	98.2 (7.2)	98.2 (7.4)	98.3 (1.7)
pH at test end (mean, SD)	7.2 (0.1)	7.2 (0.1)	7.3 (0.1)	7.2 (0.1)	7.2 (0.1)	7.3 (0.1)
Arterial partial pressure of O_2 (PaO_2) at test end (mmHg; mean, SD)	290.7 (125.3)	290.0 (124.9)	306.3 (140.1)	293.2 (122.0)	292.3 (121.0)	312.3 (146.1)
Arterial partial pressure of CO_2 ($PaCO_2$) at test end (mmHg; mean, SD)	72.1 (10.5)	72.8 (9.6)	57.1 (16.6)	72.1 (10.6)	72.8 (9.7)	57.1 (16.6)
Arterial saturation of O_2 (SaO_2) at test end (%; mean, SD)	97.0 (9.9)	96.9 (10.1)	99.1 (1.0)	97.5 (7.6)	97.4 (7.8)	99.1 (1.0)
Test stop time (mins; mean, SD)	11.9 (4.3)	12.5 (3.8)	5.1 (5.0)	11.9 (4.3)	12.5 (3.7)	5.2 (4.9)
Respiratory effort (n, %)	19 (6.7%)	0 (0.0%)	19 (24.4%)	20 (7.1%)	0 (0.0%)	20 (25.6%)
Clinical evaluation verdict (n, %)						

© 2025 Chassé M et al. *JAMA Neurology*.

Compatible with death by neurologic criteria	204 (72.3%)	204 (100.0%)	0 (0.0%)	204 (72.3%)	204 (100.0%)	0 (0.0%)
Evaluating clinician (n, %)						
Neurologist	5 (1.8%)	3 (1.5%)	2 (2.6%)	19 (6.7%)	10 (4.9%)	9 (11.5%)
Neurosurgeon	0 (0.0%)	0 (0.0%)	0 (0.0%)	0 (0.0%)	0 (0.0%)	0 (0.0%)
Intensivist	269 (95.4%)	194 (95.1%)	75 (96.2%)	233 (82.6%)	180 (88.2%)	53 (67.9%)
Resident (last year of specialty training)	6 (2.1%)	5 (2.5%)	1 (1.3%)	19 (6.7%)	11 (5.4%)	8 (10.3%)
Other	2 (0.7%)	2 (1.0%)	0 (0.0%)	11 (3.9%)	3 (1.5%)	8 (10.3%)
Clinician aware of study CT-perfusion or CT-angiography result prior to clinical examination	3 (1.1%)	2 (1.0%)	1 (1.3%)	1 (0.4%)	1 (0.5%)	0 (0.0%)
Clinician aware of any other ancillary investigation performed prior to clinical examination	18 (6.4%)	13 (6.4%)	5 (6.4%)	16 (5.7%)	12 (5.9%)	4 (5.1%)

[a]Adjudicated, blinded to neuroimaging results and included.

© 2025 Chassé M et al. *JAMA Neurology*.

eTable 4. Violations – Descriptions

This tables describe identified violations post enrolment but were kept in the analysis after blinded adjudication.

	**Entire cohort**	**Deceased**	**Alive**
Violations and deviations (n)	14	11	3
Hypothermia < 34°C[a] (n, %)	9 (4.1%)	9 (5.2%)	0 (0.0%)
Attending physician disagrees to conduct a required apnea test[b] (n, %)	1 (0.5%)	1 (0.6%)	0 (0.0%)
Reference standard (clinical examination) not completed (n, %)	4 (1.8%)	1 (0.6%)	3 (6.5%)

a Occurring between enrollment and study procedures (ancillary investigation and standard reference).

b A contemporary apnea test was completed for clinical reasons.

© 2025 Chassé M et al. *JAMA Neurology*.

eTable 5. Clinical Outcomes

	Entire cohort N=282	Deceased n=204	Alive n=78
Vital status			
Alive at discharge from intensive care unit (n, %)			
Yes	7 (2.5%)	0 (0.0%)	7 (9.0%)
No	275 (97.5%)	204 (100.0%)	71 (91.0%)
Alive at discharge from hospital (n, %)			
Yes	6 (2.1%)	0 (0.0%)	6 (7.7%)
No	1 (0.4%)	0 (0.0%)	1 (1.3%)
Not applicable	275 (97.5%)	204 (100.0%)	71 (91.0%)
Discharge location (n, %)			
Home	0 (0.0%)	0 (0.0%)	0 (0.0%)
Palliative care facility	1 (0.4%)	0 (0%)	1 (1.3%)
Other hospital intensive care unit	3 (1.1%)	0 (0%)	3 (3.8%)
Long-term care facility	0 (0.0%)	0 (0.0%)	0 (0.0%)
Rehabilitation center	0 (0.0%)	0 (0.0%)	0 (0.0%)
Other	2 (0.7%)	0 (0.0%)	2 (2.6%)
Not applicable	276 (97.9%)	204 (100.0%)	72 (92.3%)
Type of death (n, %)			
Death determination by circulatory criteria	52 (18.4%)	0 (0.0%)	52 (66.7%)
Death determination by neurologic criteria	223 (79.1%)	204 (100.0%)	19 (24.4%)
Not applicable	7 (2.5%)	0 (0.0%)	7 (9.0%)

© 2025 Chassé M et al. *JAMA Neurology*.

eTable 6. Ancillary Investigation-Related Adverse Events

	**Entire cohort N=282**	**Deceased n=204**	**Alive n=78**
Hypertension (n, %)	10 (3.5%)	6 (2.9%)	4 (5.1%)
Hypotension (n, %)	3 (1.1%)	3 (1.5%)	0 (0%)
New desaturation (n, %)	2 (0.7%)	1 (0.5%)	1 (1.3%)
Accidental extubation (n, %)	0 (0%)	0 (0%)	0 (0%)
New catheter dysfunction (n, %)	1 (0.4%)	1 (0.5%)	0 (0%)
Code blue (n, %)	0 (0%)	0 (0%)	0 (0%)
Other adverse events (n, %)	2 (0.7%)	1 (0.5%)	1 (1.3%)
No adverse event (n, %)	268 (95.0%)	195 (95.6%)	73 (93.6%)
Lowest ICP (mmHg; mean, SD) [a]	35.0 (32.0)	43.9 (37.0)	24.2 (21.8)
Highest ICP (mmHg; mean, SD) [a]	47.9 (36.6)	59.2 (41.2)	34.3 (25.9)

[a] Among the 22 patients with an intracranial pressure monitor

ICP: intracranial pressure.

© 2025 Chassé M et al. *JAMA Neurology*.

eTable 7. Details on False Positive Cases on Qualitative Brainstem CT-Perfusion (n=20)

These false positive cases were determined to be alive on clinical examination and had qualitative brainstem CT-perfusion results compatible with death.

Demographics	Brain injury	Delay between injury and CTP	Delay between CTP and clinical exam	Clinical exam findings consistent with life	Qualitative whole-brain CTP result	CTA result (late phase 10-point scale)	Outcome at hospital discharge
67 M	Hemorrhagic stroke	4 days	56 minutes	Cough reflex	Alive	Alive	DNC
69 M	Hemorrhagic stroke	1 day	15 minutes	Cough and pharyngeal reflexes	Alive	Alive	DNC
63 F	Hemorrhagic stroke	2 days	19 minutes	Caloric testing of right ear provoked constriction of ipsilateral pupil	Alive	Dead	DNC
60 F	Hemorrhagic stroke	25 days	60 minutes	Motor response to painful stimulus<br>Bilateral corneal, cough and pharyngeal reflexes<br>Breathing during apnea test	Alive	Alive	WLST and DCC
34 F	Anoxic brain injury	5 days	80 minutes	Motor response to painful stimulus<br>Breathing during apnea test	Alive	Alive	DNC
61 M	Hemorrhagic stroke	4 days	60 minutes	Cough and pharyngeal reflexes<br>Breathing during apnea test	Alive	Alive	DNC
60 M	Traumatic brain injury	6 days	40 minutes	Motor response to painful stimulus	Dead	Dead	WLST and DCC
27 M	Anoxic brain injury	3 days	99 minutes	Motor response to painful stimulus	Dead	Alive	DNC
59 F	Anoxic brain injury	4 days	15 minutes	Breathing during apnea test on ventilator. Clinician refused t-tube.	Dead	Dead	DNC
40 M	Hemorrhagic stroke	4 days	106 minutes	Cough and pharyngeal reflexes	Alive	Dead	WLST and DCC
76 M	Hemorrhagic stroke	1 day	24 minutes	Motor response to painful stimulus<br>Cough reflex	Alive	Alive	WLST and DCC

© 2025 Chassé M et al. *JAMA Neurology*.

39 M	Hemorrhagic stroke	3 days	7 minutes	Motor response to painful stimulus<br>Bilateral pupillary reflex, unilateral corneal reflex, cough and pharyngeal reflexes	Alive	Alive	WLST and DCC
60 F	Hemorrhagic stroke	1 day	180 minutes	Motor response to painful stimulus	Dead	Dead	DNC
62 M	Anoxic brain injury	4 days	21 minutes	Motor response to painful stimulus<br>Bilateral pupillary, bilateral corneal, bilateral oculo-vestibular, cough and pharyngeal reflexes	Alive	Alive	WLST and DCC
58 F	Traumatic brain injury	2 days	2 minutes	Motor response to painful stimulus<br>Cough and pharyngeal reflexes	Alive	Alive	WLST and DCC
43 M	Anoxic brain injury	4 days	38 minutes	Breathing during apnea test	Dead	Dead	DNC
65 F	Hemorrhagic stroke	3 days	51 minutes	Motor response to painful stimulus<br>Cough reflex	Alive	Alive	WLST and DCC
55 F	Hemorrhagic stroke	2 days	27 minutes	Failed apnea test (hypotension) and declared alive based on incomplete exam	Dead	Dead	DNC
69 M	Hemorrhagic stroke	1 day	14 minutes	Cough and pharyngeal reflexes<br>Triggering the ventilator	Alive	Alive	DNC
67 M	Ischemic stroke	1 day	45 minutes	Breathing during apnea test	Alive	Alive	DNC

CTP: CT-perfusion; CTA: CT-angiography; M: male; F: female; DNC: death by neurologic criteria; WLST: withdrawal of life-sustaining therapies; DCC: death by circulatory criteria

© 2025 Chassé M et al. *JAMA Neurology*.

eTable 8. Details on False Negative Cases on Qualitative Brainstem CT-Perfusion (n=3)

These false negative cases were determined to be dead on clinical examination and had qualitative brainstem CT-perfusion results incompatible with death (i.e. preserved perfusion).

Demographics	Brain injury	Delay between injury and CTP	Delay between CTP and clinical exam	Qualitative whole-brain CTP result	CTA result (late phase 10-point scale)	Outcome at hospital discharge
55 F	Hemorrhagic stroke	3 days	58 minutes	Alive	Alive	DNC
63 M	Hemorrhagic stroke	1 day	9 minutes	Alive	Alive	DNC
25 F	Traumatic brain injury	3 days	9 minutes	Alive	Alive	DNC

CTP: CT-perfusion; CTA: CT-angiography; M: male; F: female; DNC: death by neurologic criteria.

© 2025 Chassé M et al. *JAMA Neurology*.

eTable 9. Details on False Positive Cases on Qualitative Whole-Brain CT-Perfusion (n=6)

These false positive cases were determined to be alive on clinical examination and had qualitative whole-brain CT-perfusion results compatible with death.

**Demographics**	**Brain injury**	**Delay between injury and CTP**	**Delay between CTP and clinical exam**	**Clinical exam findings deemed consistent with life**	**Qualitative brainstem CTP result**	**CTA result (late phase 10-point scale)**	**Outcome at hospital discharge**
60 M	Traumatic brain injury	6 days	40 minutes	Motor response to painful stimulus	Dead	Dead	WLST and DCC
27 M	Anoxic brain injury	3 days	99 minutes	Motor response to painful stimulus	Dead	Alive	DNC
59 F	Anoxic brain injury	4 days	15 minutes	Breathing during apnea test on ventilator. Clinician refused t-tube.	Dead	Dead	DNC
60 F	Hemorrhagic stroke	1 day	180 minutes	Motor response to painful stimulus	Dead	Dead	DNC
43 M	Anoxic brain injury	4 days	38 minutes	Breathing during apnea test	Dead	Dead	DNC
55 F	Hemorrhagic stroke	2 days	27 minutes	Failed apnea test (hypotension) and declared alive based on incomplete exam	Dead	Dead	DNC

CTP: CT-perfusion; CTA: CT-angiography; M: male; F: female; DNC: death by neurologic criteria; WLST: withdrawal of life-sustaining therapies; DCC: death by circulatory criteria

© 2025 Chassé M et al. *JAMA Neurology*.

eTable 10. Details on False Negative Cases on Qualitative Whole-Brain CT-Perfusion (n=13)

These false negative cases were determined to be dead on clinical examination and had qualitative whole-brain CT-perfusion results incompatible with death (i.e. preserved perfusion).

**Demographics**	**Brain injury**	**Delay between injury and CTP**	**Delay between CTP and clinical exam**	**Qualitative brainstem CTP result**	**CTA result (late phase 10-point scale)**	**Outcome at hospital discharge**
67 F	Hemorrhagic stroke	0 days	19 minutes	Dead	Alive	DNC
76 F	Hemorrhagic stroke	2 days	78 minutes	Dead	Alive	DNC
76 F	Ischemic stroke	6 days	100 minutes	Dead	Alive	DNC
58 F	Hemorrhagic stroke	1 day	29 minutes	Dead	Dead	DNC
55 F	Hemorrhagic stroke	3 days	58 minutes	Alive	Alive	DNC
76 M	Hemorrhagic stroke	1 day	20 minutes	Dead	Alive	DNC
38 M	Hemorrhagic stroke	1 day	40 minutes	Dead	Alive	DNC
51 M	Hemorrhagic stroke	2 days	40 minutes	Dead	Alive	DNC
63 M	Hemorrhagic stroke	1 day	9 minutes	Alive	Alive	DNC
25 F	Traumatic brain injury	3 days	9 minutes	Alive	Alive	DNC
51 M	Ischemic stroke	4 days	32 minutes	Dead	Alive	DNC
63 M	Traumatic brain injury	3 days	67 minutes	Dead	Dead	DNC
79 F	Hemorrhagic stroke	1 day	44 minutes	Dead	Dead	DNC

CTP: CT-perfusion; CTA: CT-angiography; M: male; F: female; DNC: death by neurologic criteria.

© 2025 Chassé M et al. *JAMA Neurology*.

eTable 11. Sensitivity Analysis: Exclusion of Patients Considered Alive Based on Peripheral Movements Alone (n = 279)

Ancillary investigation	Sensitivity (%, 95% CI)	Specificity (%, 95% CI)	Accuracy (%, 95% CI)	Positive predictive value (%, 95% CI)	Negative predictive value (%, 95% CI)	Positive likelihood ratio (95% CI)	Negative likelihood ratio (95% CI)	Cohen's kappa (95% CI)
Qualitative brainstem CT-perfusion	98.5 (95.8; 99.7)	76.0 (64.7; 85.1)	92.5 (88.7; 95.3)	91.8 (87.3; 95.1)	95.0 (86.1; 99.0)	4.1 (2.7; 6.1)	0.02 (0.01; 0.06)	0.80 (0.73; 0.90)
Qualitative whole-brain CT-perfusion	93.6 (89.3; 96.6)	93.3 (85.1; 97.8)	93.5 (90.0; 96.1)	97.4 (94.1; 99.2)	84.3 (74.7; 91.4)	14.0 (6.0; 26.5)	0.07 (0.04; 0.12)	0.82 (0.75; 0.90)

© 2025 Chassé M et al. *JAMA Neurology*.
