# Supplementary material for: Computed Tomography Perfusion and Angiography for Death by Neurologic Criteria
Source: JAMA Neurol. 2025 Jun 13;82(9):932–40. doi: 10.1001/jamaneurol.2025.2375 (PMC12166499; doi:10.1001/jamaneurol.2025.2375)
Supplement: Supplement 2. — INDex Investigators and Canadian Critical Care Trials Group Members [file jamaneurol-e252375-s002.pdf]

Supplemental Online Content: Nonauthor Collaborators
*First name, last name, and suffix (if applicable) are required and will appear in PubMed.

*Group Name(s): INDex Investigators and Canadian Critical Care Trials Group							
***First Name and Middle Initial(s)**	***Last Name**	***Suffix (eg, Jr, III)**	Academic Degrees	Institution	Location (city, state/province, country)	Role or Contribution, eg, chair, principal investigator	Group (if more than 1 Group listed in the byline) and/or Subgroup (eg, Steering Committee)
Pierre	Aslanian		MD	CHU de Montreal	Montreal, Québec, Canada	Site Investigator	INDex Investigators
Sylvain	Belisle		MD	CHU de Montreal	Montreal, Québec, Canada	Site Investigator	INDex Investigators
François Martin	Carrier		MD, PhD	CHU de Montreal	Montreal, Québec, Canada	Site Investigator	INDex Investigators, Canadian Critical Care Trials Group
Pierre-Marc	Chagnon		MD	CHU de Montreal	Montreal, Québec, Canada	Site Investigator	INDex Investigators
Annick	Chatillon		MD	CHU de Montreal	Montreal, Québec, Canada	Site Investigator	INDex Investigators
Daniel	Corsilli		MD	CHU de Montreal	Montreal, Québec, Canada	Site Investigator	INDex Investigators
Julie	Cousineau		MD	CHU de Montreal	Montreal, Québec, Canada	Site Investigator	INDex Investigators
Tudor	Costacescu		MD	CHU de Montreal	Montreal, Québec, Canada	Site Investigator	INDex Investigators
Marc-Jacques	Dubois		MD	CHU de Montreal	Montreal, Québec, Canada	Site Investigator	INDex Investigators
Andréa	Gagnon-Hamelin			CHU de Montreal	Montreal, Québec, Canada	Research Coordinator	INDex Investigators
Mélissa	Gagnon-Hamelin			CHU de Montreal	Montreal, Québec, Canada	Research Coordinator	INDex Investigators
Martin	Girard		MD, MSc	CHU de Montreal	Montreal, Québec, Canada	Site Investigator	INDex Investigators
François	Guilbert		MD	CHU de Montreal	Montreal, Québec, Canada	Site Investigator (Radiology)	INDex Investigators
Jean-Gilles	Guimond		MD	CHU de Montreal	Montreal, Québec, Canada	Site Investigator	INDex Investigators
Christophe	Kolan		MD, MSc	CHU de Montreal	Montreal, Québec, Canada	Site Investigator	INDex Investigators
Dominique	Lafrance		MD	CHU de Montreal	Montreal, Québec, Canada	Site Investigator	INDex Investigators

Supplemental Online Content: Nonauthor Collaborators
*First name, last name, and suffix (if applicable) are required and will appear in PubMed.

***First Name and Middle Initial(s)**	***Last Name**	***Suffix (eg, Jr, III)**	Academic Degrees	Institution	Location (city, state/province, country)	Role or Contribution, eg, chair, principal investigator	Group (if more than 1 Group listed in the byline) and/or Subgroup (eg, Steering Committee)
Francesca	Lamothe			CHU de Montreal	Montreal, Québec, Canada	Research Coordinator	INDex Investigators
Jean-François	Lizé		MD	CHU de Montreal	Montreal, Québec, Canada	Site Investigator	INDex Investigators
Nicholas	Robillard		MD, MSc	CHU de Montreal	Montreal, Québec, Canada	Site Investigator	INDex Investigators
Catalina	Sokoloff		MD	CHU de Montreal	Montreal, Québec, Canada	Site Investigator	INDex Investigators
David	Bellemare			CHU de Québec–Université Laval	Québec City, Québec, Canada	Research Coordinator	INDex Investigators
Jean-Luc	Gariépy		MD	CHU de Québec–Université Laval	Québec City, Québec, Canada	Site Investigator (Radiology)	INDex Investigators
Stéphanie	Grenier			CHU de Québec–Université Laval	Québec City, Québec, Canada	Research Coordinator	INDex Investigators
Gabrielle	Guilbaut			CHU de Québec–Université Laval	Québec City, Québec, Canada	Research Coordinator	INDex Investigators
Marie-Pier	Bouchard			CHU de Sherbrooke	Sherbrooke, Québec, Canada	Research Coordinator	INDex Investigators
Élaine	Carbonneau			CHU de Sherbrooke	Sherbrooke, Québec, Canada	Research Coordinator	INDex Investigators
Jean	Chénard		MD	CHU de Sherbrooke	Sherbrooke, Québec, Canada	Site Investigator (Radiology)	INDex Investigators
François	Lamontagne		MD, MSc	CHU de Sherbrooke	Sherbrooke, Québec, Canada	Site Investigator	INDex Investigators, Canadian Critical Care Trials Group
Charles	St-Arnaud		MD	CHU de Sherbrooke	Sherbrooke, Québec, Canada	Site Investigator	INDex Investigators
Cassidy	Codan			Foothills Medical Center	Calgary, Alberta, Canada	Research Coordinator	INDex Investigators
Olesya	Dmitrieva			Foothills Medical Center	Calgary, Alberta, Canada	Research Coordinator	INDex Investigators
Muneer	Eesa		MD	Foothills Medical Center	Calgary, Alberta, Canada	Site Investigator (Radiology)	INDex Investigators
Lily	Guan			Foothills Medical Center	Calgary, Alberta, Canada	Research Coordinator	INDex Investigators
Julie	Kromm		MD	Foothills Medical Center	Calgary, Alberta, Canada	Site Investigator	INDex Investigators
Lori	Hand			Hamilton Health Sciences	Hamilton, Ontario, Canada	Research Coordinator	INDex Investigators

Supplemental Online Content: Nonauthor Collaborators
*First name, last name, and suffix (if applicable) are required and will appear in PubMed.

***First Name and Middle Initial(s)**	***Last Name**	***Suffix (eg, Jr, III)**	Academic Degrees	Institution	Location (city, state/province, country)	Role or Contribution, eg, chair, principal investigator	Group (if more than 1 Group listed in the byline) and/or Subgroup (eg, Steering Committee)
Draga	Jichici			Hamilton Health Sciences	Hamilton, Ontario, Canada	Research Coordinator	INDex Investigators
Arun	Mensinkai			Hamilton Health Sciences	Hamilton, Ontario, Canada	Research Coordinator	INDex Investigators
Andrée-Anne	Pistono		MD	Hôpital Maisonneuve-Rosemont	Montreal, Québec, Canada	Site Investigator (Radiology)	INDex Investigators
Danaë	Tassy			Hôpital Maisonneuve-Rosemont	Montreal, Québec, Canada	Research Coordinator	INDex Investigators
Tracy	Boyd			Kingston Health Sciences Centre – KGH	Kingston, Ontario, Canada	Research Coordinator	INDex Investigators
Miranda	Hunt			Kingston Health Sciences Centre – KGH	Kingston, Ontario, Canada	Research Coordinator	INDex Investigators
Omar	Islam		MD	Kingston Health Sciences Centre – KGH	Kingston, Ontario, Canada	Site Investigator	INDex Investigators
Tracey	Bental			London Health Sciences	London, Ontario, Canada	Research Coordinator	INDex Investigators
Michael	Jurkiewicz		MD	London Health Sciences	London, Ontario, Canada	Site Investigator (Radiology)	INDex Investigators
Claudio	Martin		MD	London Health Sciences	London, Ontario, Canada	Site Investigator	INDex Investigators, Canadian Critical Care Trials Group
Marat	Slessarev		MD	London Health Sciences	London, Ontario, Canada	Site Investigator	INDex Investigators, Canadian Critical Care Trials Group
Siddiqui	Faisal		MD	Health Sciences Centre	Winnipeg, Manitoba, Canada	Site investigator	INDex Investigators
Khunsa	Faiz		MD	Health Sciences Centre	Winnipeg, Manitoba, Canada	Research Coordinator	INDex Investigators
Nicole	Marten			Health Sciences Centre	Winnipeg, Manitoba, Canada	Research Coordinator	INDex Investigators
Maggie	Wilson			Health Sciences Centre	Winnipeg, Manitoba, Canada	Research Coordinator	INDex Investigators
Susan	Alcock		MSc	Health Sciences Centre	Winnipeg, Manitoba, Canada	Research Coordinator	INDex Investigators
Josie	Campisi			McGill University Health Center	Montreal, Québec, Canada	Research Coordinator	INDex Investigators

Supplemental Online Content: Nonauthor Collaborators
*First name, last name, and suffix (if applicable) are required and will appear in PubMed.

***First Name and Middle Initial(s)**	***Last Name**	***Suffix (eg, Jr, III)**	Academic Degrees	Institution	Location (city, state/province, country)	Role or Contribution, eg, chair, principal investigator	Group (if more than 1 Group listed in the byline) and/or Subgroup (eg, Steering Committee)
Jeffrey	Chankowsky		MD	McGill University Health Center	Montreal, Québec, Canada	Site Investigator (Radiology)	INDex Investigators
Kosar	Khwaja		MD	McGill University Health Center	Montreal, Québec, Canada	Site Investigator	INDex Investigators
Raham	Rahgoshai			McGill University Health Center	Montreal, Québec, Canada	Research Coordinator	INDex Investigators
Deborah J	Cook		MD, MSc	McMaster University	Hamilton, Ontario, Canada	CCCTG peer review	Canadian Critical Care Trials Group
Bram	Rochwerg		MD, MSc	McMaster University	Hamilton, Ontario, Canada	CCCTG peer review	Canadian Critical Care Trials Group
Erin	Cole			Montreal Neurological Institute	Montreal, Québec, Canada	Research Coordinator	INDex Investigators
Rick	Sanchez			Montreal Neurological Institute	Montreal, Québec, Canada	Research Coordinator	INDex Investigators
Catherine	Therrien			Montreal Neurological Institute	Montreal, Québec, Canada	Research Coordinator	INDex Investigators
Gilbert	Yip			Montreal Neurological Institute	Montreal, Québec, Canada	Research Coordinator	INDex Investigators
Valerie	Barette			QEII Health Sciences Center	Halifax, Nova Scotia, Canada	Research Coordinator	INDex Investigators
Laura	Magennis			QEII Health Sciences Center	Halifax, Nova Scotia, Canada	Research Coordinator	INDex Investigators
Matthias	Schmidt		MD	QEII Health Sciences Center	Halifax, Nova Scotia, Canada	Radiologist	INDex Investigators
Robert	Vandorpe		MD	QEII Health Sciences Center	Halifax, Nova Scotia, Canada	Site Investigator (Radiology)	INDex Investigators
Aditya	Bharatha		MD	St-Michael’s Hospital	Toronto, Ontario, Canada	Site Investigator (Radiology)	INDex Investigators
Imrana	Khalid			St-Michael’s Hospital	Toronto, Ontario, Canada	Research Coordinator	INDex Investigators
Gyan	Sandhu			St-Michael’s Hospital	Toronto, Ontario, Canada	Research Coordinator	INDex Investigators
Marlene	Santos			St-Michael’s Hospital	Toronto, Ontario, Canada	Research Coordinator	INDex Investigators
Santanu	Chakraborty		MD	The Ottawa Hospital	Ottawa, Ontario, Canada	Site Investigator (Radiology)	INDex Investigators
Jessica	Haines			The Ottawa Hospital	Ottawa, Ontario, Canada	Research Coordinator	INDex Investigators
Greg	Knoll		MD, MSc	The Ottawa Hospital	Ottawa, Ontario, Canada	Site Investigator	INDex Investigators

Supplemental Online Content: Nonauthor Collaborators
*First name, last name, and suffix (if applicable) are required and will appear in PubMed.

***First Name and Middle Initial(s)**	***Last Name**	***Suffix (eg, Jr, III)**	Academic Degrees	Institution	Location (city, state/province, country)	Role or Contribution, eg, chair, principal investigator	Group (if more than 1 Group listed in the byline) and/or Subgroup (eg, Steering Committee)
Doug	McGuire			The Ottawa Hospital	Ottawa, Ontario, Canada	Data Management	INDex Investigators
Rebecca	Porteous			The Ottawa Hospital	Ottawa, Ontario, Canada	Research Coordinator	INDex Investigators
Irene	Watpool			The Ottawa Hospital	Ottawa, Ontario, Canada	Research Coordinator	INDex Investigators
Noha	Aref			William Osler Health Center	Brampton, Ontario, Canada	Research Coordinator	INDex Investigators
Vinayak	Lad			William Osler Health Center	Brampton, Ontario, Canada	Research Coordinator	INDex Investigators
Rosa Myrna	Marticorena			William Osler Health Center	Brampton, Ontario, Canada	Research Coordinator	INDex Investigators
Marc	Ossip		MD	William Osler Health Center	Brampton, Ontario, Canada	Site Investigator (Radiology)	INDex Investigators
