# Supplementary material for: Computed Tomography Perfusion and Angiography for Death by Neurologic Criteria
Source: JAMA Neurol. 2025 Jun 13;82(9):932–40. doi: 10.1001/jamaneurol.2025.2375 (PMC12166499; doi:10.1001/jamaneurol.2025.2375)
Supplement: Supplement 3. — Data Sharing Statement [file jamaneurol-e252375-s003.pdf]

Data Sharing Statement

Chassé. Computed Tomography Perfusion and Angiography for Death by Neurologic Criteria. *JAMA Neurol*. Published June 13, 2025. doi:10.1001/jamaneurol.2025.2375

Data
Data available: No

Additional Information
Explanation for why data not available: The sharing of individual patient data is restricted in accordance with applicable local laws of Canada. However, access to the full dataset, including individual patient data, may be possible for audit purposes or for secondary studies if they align with the orignal study protocol and informed patient consent. Such access would be contingent upon a formal data sharing agreement, ethical review board approval, and will be facilitated within a secure, controlled environment.
